# Design of a Mobile App and a Clinical Trial Management System for Cognitive Health and Dementia Risk Reduction: User-Centered Design Approach

**DOI:** 10.2196/66660

**Published:** 2025-07-02

**Authors:** Hannes Hilberger, Bianca Buchgraber-Schnalzer, Simone Huber, Theresa Weitlaner, Markus Bödenler, Alara Abaci, Jeroen Bruinsma, Ana Diaz, Anna Giulia Guazzarini, Jenni Lehtisalo, Seungjune Lee, Vasileios Loukas, Francesca Mangialasche, Patrizia Mecocci, Tiia Ngandu, Anna Rosenberg, Elisabeth Stögmann, Konsta Valkonen, Elena Uhlik, Helena Untersteiner, Laura Kneß, Helmut Ahammer, Sten Hanke

**Affiliations:** 1 Institute of eHealth University of Applied Sciences - FH JOANNEUM Graz Austria; 2 Gottfried Schatz Research Center, Division of Medical Physics and Biophysics Medical University of Graz Graz Austria; 3 Division of Clinical Geriatrics, Center for Alzheimer Research, Department of Neurobiology, Care Sciences and Society Karolinska Institutet Stockholm Sweden; 4 Department of Health Promotion, Care and Public Health Research Institute Maastricht University Maastricht The Netherlands; 5 Alzheimer Europe Luxembourg Luxembourg; 6 Division of Gerontology and Geriatrics, Department of Medicine and Surgery University of Perugia Perugia Italy; 7 Division of Clinical Geriatrics, Department of Neurobiology, Care Sciences and Society Karolinska Institutet Stockholm Sweden; 8 Lifestyles and Living Environments Unit, Department of Public Health Finnish Institute for Health and Welfare Helsinki Finland; 9 Department of Neurology Medical University of Vienna Vienna Austria; 10 Comprehensive Center for Clinical Neurosciences and Mental Health Medical University of Vienna Vienna Austria; 11 Department of Materials Science and Engineering, Unit of Medical Technology and Intelligent Information Systems University of Ioannina Ioannina Greece; 12 Biomedical Research Institute Foundation for Research and Technology–Hellas Ioannina Greece; 13 Medical Unit Aging, Theme Inflammation and Aging Karolinska Institutet Stockholm Sweden; 14 Institute of Public Health and Clinical Nutrition University of Eastern Finland Kuopio Finland; 15 Institute of Clinical Medicine University of Eastern Finland Kuopio Finland

**Keywords:** clinical trial, medical informatics, health information systems, eHealth, mobile health, mHealth, mobile apps, dementia, cognitive decline, prevention, multidomain interventions, artificial intelligence, AI

## Abstract

**Background:**

The rising prevalence of dementia is a major concern, with approximately 45% of cases linked to 14 modifiable risk factors. The European project LETHE aims to develop a personalized digital intervention model to delay or prevent cognitive decline through risk factor management.

**Objective:**

The objective of our study was to design a clinical trial platform for older individuals at risk of cognitive decline, including a mobile app for study participants and a clinical trial management system (CTMS) for health professionals.

**Methods:**

Using a user-centered design approach, workshops and feedback rounds involved potential participants representing the target group and professionals. The LETHE app’s usability was assessed among 156 older adults enrolled in a 2-year multinational randomized controlled trial evaluating the feasibility of a digitally supported lifestyle program for dementia risk reduction. The randomized controlled trial is currently ongoing; the System Usability Scale (SUS) was administered 1 month after baseline to map first user experiences. Feedback on the LETHE CTMS was collected from 21 users.

**Results:**

Of the 78 participants in the trial intervention group, 66 (85%) provided responses for the mobile app, with a median SUS score of 70 (IQR 55-82). Within the control group, 73% (57/78) of responses were received, with a median SUS score of 73 (IQR 63-90). For the CTMS, we received 71% (15/21) of responses, and the feedback was mostly positive. A ranking of the features that could be considered beyond state of the art showed that the integration of personalized activities (mean 2.23, SD 1.17) and real-time appointments (mean 2.46, SD 1.51) were considered the most novel ones.

**Conclusions:**

The LETHE app and CTMS were developed to support a personalized digital intervention method within a study involving 156 participants. Limitations include participants having digital literacy and internet access, potentially impacting the generalizability of the findings. Despite these limitations, positive feedback and high usability scores suggest promising potential for the LETHE app and CTMS in supporting personalized interventions to prevent cognitive decline in older adults.

## Introduction

### Background

#### Overview

The rising prevalence of dementia, driven by sociodemographic changes, constitutes a significant global health challenge. Projections indicate a notable rise from 57 million affected individuals in 2019 to an anticipated 153 million by 2050 [1]. Notably, the Lancet Commission’s report underscores that approximately 45% of all cases of dementia are associated with 14 potentially modifiable risk factors at different phases of the life span [2], and diet has also been suggested as an additional factor [3]. Previous multidomain intervention studies have demonstrated their positive impact on cognitive performance while simultaneously targeting various lifestyle domains, including diet, physical activity, cognitive training, management of cardiovascular risk factors, or social interaction [4-6].

Information and communications technology (ICT) solutions such as apps running on phones and tablets, as well as wearable devices that collect user-generated health data (digital biomarkers) in an automated way, could potentially help provide digitally supported interventions for a broader audience and groups at risk of cognitive decline. At the same time, these technologies can collect data to monitor progress and adherence to lifestyle interventions. Other research projects have shown that digital interventions can be effective and feasible for older adults, but this depends on several factors such as digital literacy, usability of the technology, and the design of the study with regard to human support (hybrid intervention) [7-9].

The Horizon 2020 LETHE project [10] has been initiated to provide such a personalized digital intervention model and components for reducing risk of developing cognitive decline by evolving the successful Finnish Geriatric Intervention Study to Prevent Cognitive Impairment and Disability (FINGER) multidomain intervention model [5]. Leveraging ICT-based methods and guided in-person as well as remote sessions, LETHE aims to prevent cognitive decline in at-risk older individuals by providing personalized feedback and intervention methods.

The LETHE project focuses on the aforementioned modifiable risk factors grouped into the following lifestyle domains: “Physical Activity,” “Nutrition,” “Cognitive Training,” “Management of vascular/metabolic risk factors,” “Sleep/Relaxation,” and “Social Activity.”

The LETHE study encompasses a currently ongoing 2-year randomized controlled multicenter, parallel-group feasibility trial (ClinicalTrials.gov; NCT05565170) involving 156 participants at risk of cognitive decline distributed across clinical centers in Austria (Medical University of Vienna), Finland (Finnish Institute for Health and Welfare), Sweden (Karolinska Institute), and Italy (University of Perugia). Participants were equally randomized to one of the study groups (intervention and control), both supported by digital tools (mobile app and smartwatch). At baseline, participants had a mean age of 68.8 (SD 4.5) years, with an average of 14.9 (SD 3.1) years of education. A total of 64.7% of the participants were women. Notably, most participants (84%) used their smartphones at least 6 times per day. Two-thirds of the participants had previous experience using their smartphones for health tracking, and approximately 48% had used lifestyle-related apps. In addition, 63% of the participants reported using the internet for eHealth-related tasks such as booking physician appointments or checking test results [11].

To provide intervention components and continuously collect data from the trial participants, a LETHE smartphone app was designed and implemented. Clinical data, including blood test results, are entered into the LETHE clinical trial management system (CTMS), which was also developed during the course of the project, by health professionals at each visit. The CTMS was developed to ensure seamless integration with the app, allowing all participant-entered data to be displayed for health professionals to review and respond accordingly. Furthermore, data and services from third parties have been integrated, including cognitive training data and data from Fitbit devices, which track steps, sleep, and physical activity. The feasibility of other novel technologies (audio glasses and a robot that reflect the content of the smartphone app) is being investigated in a substudy.

This study aimed to describe the design and implementation of the LETHE technical components, which were carefully developed through a joint process involving potential users, clinical experts, and designers and technicians.

#### Hybrid Intervention Approach

LETHE adopts a hybrid intervention (blended therapies) approach, seamlessly integrating digital intervention sessions with in-person and group sessions led by a trained coach (eg, physiotherapist or nutritionist). This comprehensive strategy, meticulously managed by health professionals, is in harmony with the study’s mobile app and CTMS concept.

The literature on hybrid interventions for older adults at risk of cognitive decline, incorporating technology with coaching and peer sessions in comparison to stand-alone technology interventions, is still developing and has so far mainly focused on already symptomatic individuals. Nevertheless, emerging studies indicate that fostering social connection and interaction can be pivotal for success in dementia interventions [12]. Digital health platforms, easily accessible via mobile technology, can be efficient and cost-effective tools for dementia prevention. They not only offer individualized cognitive training but can also provide an engaging user experience [13,14]. The currently ongoing prevention of dementia using mobile phone applications (PRODEMOS) study creates an evidence-based dementia prevention strategy using mobile health (mHealth) accessible to at-risk individuals [15]. In contrast, Maintain Your Brain was a completely online lifestyle intervention targeting modifiable risk factors for dementia that was delivered to the participants via a web interface [16]. Wesselman et al [17] conducted a comprehensive overview of web-based multidomain lifestyle programs, combining it with a meta-analysis to evaluate their effectiveness. Through a systematic literature research, they collected a wide range of web-based programs.

Other studies have provided app-supported self-regulation for older adults based on self-determination theory, with a tablet facilitating tailored exercise programs and playing a key role in action planning and execution of behaviors [18]. The involvement of a personal coach was pivotal, adapting exercises to individual preferences and providing motivating remote monitoring to empower older adults to enhance physical activity levels at home. The experience conveyed in the study by Mehra et al [18] emphasizes the value of the personal coach, particularly during the initial phases of goal setting, behavior execution, and evaluation in self-regulation to achieve specific goals by guiding one’s own behavior. Their findings indicate that the availability of a personal coach remains crucial even when technology supports the intervention. Nevertheless, concerns about adherence and safety arose in the absence of instructor guidance [18].

The success of this hybrid intervention approach could be further supported by the seamless interaction of a mobile app and a web-based CTMS for professionals. This integrated system allows professionals to interact with and monitor patients’ development, enabling timely interventions when needed.

#### Digital Intervention Applications

mHealth apps are increasingly used by individuals to engage in health behaviors, aid in the self-management of chronic conditions, or enact preventive measures [19]. These apps hold promise in personalizing and tailoring behavior change interventions based on real-time data, thereby enhancing health outcomes [20]. Another crucial consideration is the necessity for co-designing apps in collaboration with the end users and health professionals from the relevant sector [21]. This is particularly vital for older adults as their participation in the technological design process is pivotal for their acceptance and adoption of the technology [22].

Digital interventions, whether delivered through mHealth apps or computers, are subject to evaluation in randomized controlled trials (RCTs) targeting various conditions such as psychotic disorders [23,24], mental health [25-28], eating disorders [29], diabetes [30], chronic pain [31], insomnia [32], or speech disorders [33]. These interventions often incorporate features such as audio or video content for physical activity or relaxation [23,24,26,32]; mood tracking [23,25,26]; personal tasks [23,25,31]; questionnaires [25,27,29,31]; educational materials [23-26,32]; self-tracking of, for example, vital signs in the form of a diary [24,32]; and habit libraries for behavior changes [34].

An umbrella review comprising 48 systematic reviews concentrating on mHealth apps in RCTs across various health conditions such as diabetes or hypertension indicates the potential effectiveness of app-based health interventions [35]. This further strengthens the approach of delivering a mobile-based app for study participants.

#### CTMSs for Intervention Projects

Equally important when intervention apps are used in clinical trials or research settings is central monitoring and management by the study coordinator or a corresponding physician. Using a CTMS and the integration of electronic data capture has become commonplace to serve this purpose [36]. Such a platform should facilitate and effectively support the multidimensional data management process in clinical trials [37] throughout their phases, from participant onboarding to completion [38].

Electronic systems allow for streamlined data transfer from clinics to the CTMS, remote enrollment capabilities, greater transparency of trial conduct, timeline monitoring such as specific tasks, tracking of participant visits, enhanced research documentation, and robust reporting [36,38,39]. A CTMS presents a variety of advantages; for example, it enables research teams to access up-to-date study information and simplifies collaboration as all project members can work efficiently together on the same task [38]. Moving beyond traditional methods of data collection in clinical trials, which may involve manual completion of paper case report forms (CRFs), leads to increased accuracy in findings, enhanced productivity as all necessary elements for managing the trial are consolidated in a single location, and higher data quality and compliance while reducing the risk of bias in clinical outcomes [36]. Important considerations when selecting a CTMS include the feature set, usability, customization, and cost [38].

A systematic review of 19 research papers examining the technical features of clinical data management systems revealed that most of these systems were developed on a web-based platform to meet the individual needs of specific clinical trials within a short time frame [40]. Reportedly, such systems used in research centers showed limitations and inability to fully support the automation of all dimensions of the clinical data and workflow management process. In addition, the review found that most of the systems lacked flexibility and extensibility for further system development.

Health professionals such as study nurses, who are key members of the clinical research team and one of the targeted user groups for CTMSs, play a critical role in achieving accurate outcomes for clinical research studies. Furthermore, the effective implementation of a CTMS in RCT studies can have multiple benefits, including improved completion rates and increased fidelity while ensuring the safety of individual research participants [36].

The studies mentioned in this section collectively underscore the value of CTMSs in enhancing the efficiency and effectiveness of complex multisite intervention projects.

### Objectives

This paper describes the iterative design process and reports on the technical implementation of the LETHE app and the LETHE CTMS within the context of a multifaceted and hybrid intervention to prevent cognitive decline in older adults. By sharing the results, we aim to contribute valuable insights to the field of dementia prevention and risk factor management through personalized ICT-supported hybrid intervention methods. We collaborated intensively with end users and health professionals to design the clinical trial platform. In addition, in this study, we evaluated how the trial participants and clinicians accepted the developed applications by using subjective usability assessments. This helped us to understand whether such digital tools keep the participants more engaged and identify directions for further improvement.

## Methods

### Design of a Mobile App and Clinical Trial Platform

#### Overview

To ensure high usability, the user-centered design (UCD) approach was chosen for the LETHE app as well as for the CTMS design process. The UCD process involves the participation of end users (in our case, older individuals at risk of cognitive decline and health professionals supporting them during a preventive lifestyle intervention using the CTMS) to define key user requirements. The design approach consists of 3 main phases: analysis, design, and implementation [41].

The UCD approach encompasses 5 key principles: defining user requirements, gathering and considering feedback from users to specify requirements, involving users from the outset to evaluate design iterations, consistently adhering to UCD, and using an iterative design process [42].

**Figure 1:**
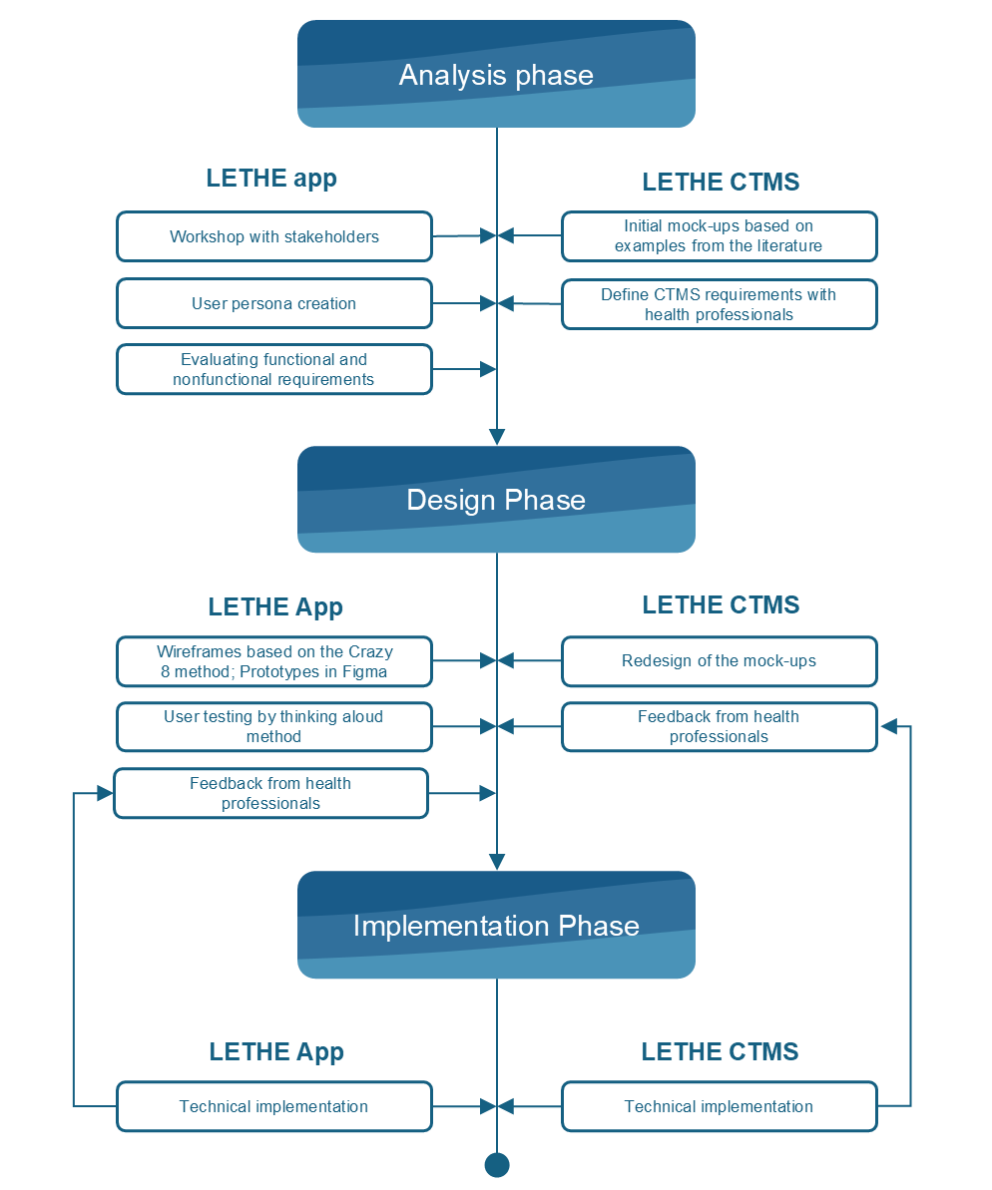
Different phases for the design and development of the LETHE app and LETHE clinical trial management system (CTMS).

Figure 1 illustrates the different design phases of the LETHE app and the LETHE CTMS.

#### Requirement Analysis and Design of the LETHE App

First, a requirement workshop, which was held online, was carried out with the goal of listing requirements for the entire LETHE ecosystem and especially for the LETHE app. A total of 21 individuals from the LETHE project, including clinicians, behavioral experts, and representatives of public involvement in dementia, were invited to the workshop.

During the workshop, 3 user personas [43] were created, which represented typical end users of the LETHE app. General requirements were evaluated via open-ended questions from a clinical view. Specific requirements such as the design or the scientific use were ranked according to importance. The functional requirements of the LETHE app included, on the one hand, the content features according to the FINGER lifestyle domains; on the other hand, general digital requirements were collected from professionals during the workshop. The design of the digital intervention was based on established behavior change theories to ensure that the app’s features were effective. We focused on understanding relevant psychological factors that influence behaviors related to dementia risk [44,45] by relying on multiple behavior change theories to achieve a comprehensive understanding [46]. We used intervention mapping elements [47] and relied on a behavior change taxonomy [48] to select strategies that would best target relevant psychological factors to promote behavior change. Methods such as self-monitoring, setting clear goals, and planning for challenges were built into the app’s features. Throughout the design process, a collaboration with health professionals and an advisory board of older adults at risk of or living with dementia was established. The results of this workshop were collected via online collaborating tools such as Mentimeter [49] and Padlet [50].

Afterward, wireframes based on the requirements and identified personas were created. During the user experience design, the method “The crazy 8” for wireframing was used to create as many screens as possible [51]. This method is a fast-sketching method to engage designers in sketching 8 distinct ideas in 8 minutes. In the next step, the wireframes were built using Figma (Figma, Inc) [52] as clickable prototypes to obtain continuous feedback from the involved health professionals.

In addition, a first user test with 4 German-speaking citizens aged between 65 and 85 years was carried out at the Department of Neurology at the Medical University of Vienna, and results were integrated during the implementation phase. One participant had a diagnosis of dementia, whereas the other 3 had no diagnosis. The user tests were conducted using the wireframes as clickable prototypes. First, participants explored the prototype using the thinking-aloud method [53]. Afterward, they were asked to perform 2 specific tasks (ie, choosing among a prepopulated list of pieces of advice for lifestyle improvement and setting them as goals for personal change and entering self-measured blood pressure values). Finally, participants provided verbal feedback and shared ideas and recommendations to further improve the prototypes.

When an implementation-ready design was obtained after the workshop and the user test, the implementation phase (including translation tasks) involved continuous feedback from professionals. Textbox 1 summarizes the phases for creating the design of the LETHE app.

**Phases for creating the design of the LETHE app, with steps 4 and 5 being recurring.**

- Workshop for creation of user personas and functional and nonfunctional requirements (step 1)
- Creation of wireframes and clickable prototypes based on the workshop (step 2)
- User testing with potential end users (step 3)
- Adaption of wireframes and implementation phase (step 4)
- Continuous improvement, modification, and development after feedback from professionals and suggestions by study participants and advisory board members (step 5)

#### Requirement Analysis and Design of the LETHE CTMS

In the initial phase of our UCD methodology, an interactive workshop dedicated to the analysis of user requirements for the LETHE CTMS was conducted. The workshop was held online via Microsoft Teams (Microsoft Corp) with 13 health professionals as participants from different study centers located in Finland, Italy, Sweden, and Austria, as well as technical partners involved in the LETHE project. The professionals of the study centers included neurologists, gerontologists, public health experts, and professionals in the field of quality of life and dementia who represented the end users of the CTMS. The aim was to collaboratively identify the essential features and functionalities required for the LETHE CTMS. The workshop used the Mural tool [54], facilitating the creation of digital whiteboards using sticky notes for brainstorming in smaller breakout rooms consisting of 3 to 4 persons. In those breakout rooms, initial design considerations were discussed. Those considerations included general thoughts on a CTMS, an overview of the participants, data entering, and an artificial intelligence (AI) risk simulation. The process behind the design of the CTMS is described in Textbox 2 [55-57].

The results of the workshop influenced the subsequent design phase, during which multiple design proposals were iteratively developed incorporating feedback from clinical professionals. Once the design phase concluded, we transitioned into the implementation phase.

**Process to design the LETHE clinical trial management system, with steps 3 and 4 being recurring steps.**

- Creation of initial mock-ups based on examples from the literature [55-57] and the study protocol of the LETHE trial for workshops together with clinical and technical partners (step 1)
- Conduction of the workshops to gather feedback and potentially missing features (step 2)
- Redesign of the mock-ups and technical implementation (step 3)
- Feedback on the redesign (step 4)

### Evaluation of the Mobile App and Clinical Trial Platform

#### Overview

This section provides a detailed overview of the methodology used to analyze user perspectives within the currently ongoing 2-year clinical feasibility trial, focusing on both the LETHE app and the LETHE CTMS (or LETHE dashboard). The aim was to gather insights on basic usability assessments, encompassing considerations such as satisfaction with the applications.

The analysis was conducted using R (version 4.3.2; R Foundation for Statistical Computing) [58]. The normality test for the data was carried out using the Shapiro-Wilk test (α=.05). To assess the difference in the System Usability Scale (SUS) scores [59] between the study groups, the Mann-Whitney *U* test was used. For pairwise comparisons of the various countries, the Kruskal-Wallis test followed by the Dunn post hoc test with Bonferroni correction was used.

#### Evaluation of the LETHE App

Older adults who participated in the LETHE RCT provided feedback within the LETHE app through a structured questionnaire, “Experiences with LETHE app,” consisting of 10 closed-ended questions based on an adapted version of the SUS, which was simplified for older adults and adults with cognitive impairment [60]. Using a Likert Scale [61] with 5 points ranging from “Strongly Disagree” to “Strongly Agree,” participants expressed subjective feelings about the frequency of LETHE app use, perceptions of app complexity, and their need for assistance or confidence in navigating the mobile app. The questionnaire can be found in Multimedia Appendix 1.

This survey will be administered at 3 distinct time points throughout the study: 1 month, 6 months, and 24 months after randomization. Participants have 4 weeks to complete the survey at each time point. This timeline allows for the observation of participant sentiments over an extended duration, offering insights into the subjective aspects of use patterns and the perceived complexity of a mobile app specifically designed for older individuals. As the trial is still ongoing, this study focused on data from the first time point. As a result, we share participants’ initial user experiences at this stage.

The LETHE app exists in 2 versions: one for the intervention group and one for the control group. Due to the limited functionality of the version for the control group, which lacks more sophisticated features such as personalized or intervention activities, the analysis was conducted separately for the intervention and control groups.

Within the LETHE trial, 156 study participants were invited to complete the questionnaire, with 78 (50%) in the intervention group and 78 (50%) in the control group. The calculated SUS was assigned a grade [62] as shown in Table 1, with “C” as the average grade and 68 as the center of the range.

**Table 1:** Interpretation of the System Usability Scale (SUS) score based on a grading scale by Sauro and Lewis [62].

SUS range	Grade	Percentile range
84.1-100	A+	96-100
80.8-84	A	90-95
78.9-80.7	A–	85-89
77.2-78.8	B+	80-84
74.1-77.1	B	70-79
72.6-74	B–	65-69
71.1-72.5	C+	60-64
65-71	C	41-59
62.7-64.9	C–	35-40
51.7-62.6	D	15-34

#### Evaluation of the LETHE CTMS

To obtain a comprehensive understanding of perspectives from relevant health professionals who used the LETHE CTMS, a 41-question online survey was conducted including both open- and closed-ended questions via Microsoft Forms (Microsoft Corp). The survey was distributed 10 months after the start of the study, and users were given 3 weeks to complete it. The primary objective was to gather feedback on the functionalities of the LETHE CTMS and assess user satisfaction with its implementation. The survey aimed to provide insights essential for mitigating common bottlenecks in the future development of similar tools. Notably, the survey was self-administered and structured into different parts, starting with a brief introduction outlining its objective before participants provided information about their role in the project. Subsequently, general inquiries about the CTMS, such as the onboarding process and overall experience, were posed. Participants then provided feedback on each functionality. Finally, recommendations, challenges, and additional comments were solicited.

All users of the LETHE CTMS were invited to participate and were informed about the survey duration.

The mixing of open- and closed-ended questions served a dual purpose. Closed-ended questions, including Likert scaling and ranking formats, enabled the quantification of participant sentiments, whereas parallel open-ended inquiries facilitated the collection of qualitative feedback. Noteworthy is the inclusion of a single ranking question designed to identify the beyond-state-of-the-art features of the LETHE CTMS.

Moreover, a 5-point Likert scale [61] was used for closed-ended questions, encompassing the following response options: “Strongly Agree,” “Agree,” “Neither Agree nor Disagree,” “Disagree,” and “Strongly Disagree.”

The structure of the survey can be found in Multimedia Appendix 2.

### Ethical Considerations

The trial has been approved by ethical committees in Austria (Ethics Committee of the Medical University of Vienna; 1392/2022), Finland (Hospital District of Helsinki and Uusimaa Ethical Committee; HUS/13675/2022), Italy (Regional Ethics Committee Umbria; 25723/22/AV), and Sweden (Swedish Ethical Review Authority; 2022-03961-01). All participants provided written informed consent before enrollment. The data used in this study for analysis were anonymized. Study participants received a wearable device, and a phone if they chose to have one. Some of those in the intervention group were additionally provided with a tablet for use during the trial.

## Results

### Design, Functionalities, and Evaluation of the LETHE App

#### Results of the Requirement Workshop and End-User Testing

The workshop together with the clinical and technical partners identified several requirements regarding the LETHE app, including the highlighting of lifestyle factors that constitute a risk or where the participant could improve. Additional observations include the LETHE app’s provision of guidelines to identify and flag study participants who are not using it. The communication between the specialists and the study participants should be simple. There was also an emphasis on data transfer and data sharing, highlighting that study participants can independently input data without professional assistance, such as for questionnaires.

**Figure 2:**
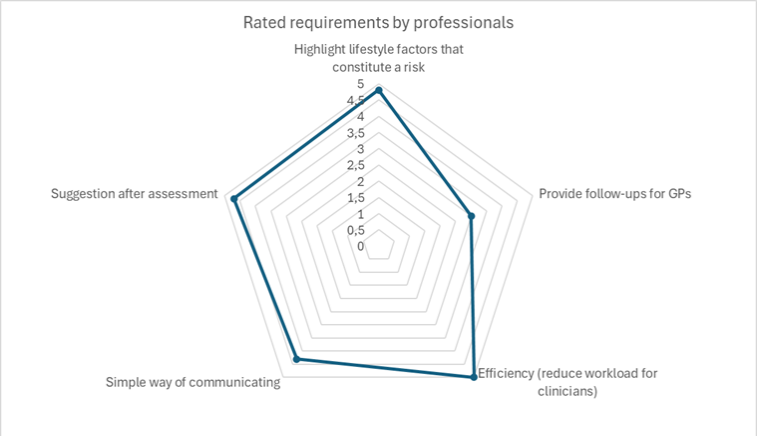
Categorized and ranked requirements for the LETHE app. GP: general practitioner.

The identified requirements were categorized and ranked via a survey. The main outcomes for the LETHE app are summarized in Figure 2. The highest-ranked requirements were to reduce the workload for professionals and highlight the lifestyle factors that constitute a risk. The least important requirement was to provide follow-ups for general practitioners. Those main findings were considered when designing the prototype.

While conducting the user evaluation with the 4 participants at the Department of Neurology at the Medical University of Vienna, several key findings were obtained. The size of the user interface elements was criticized as being too small, and the tiles were not understood as large buttons. A section about the summary of the previous week as well as the share function of third-party apps (eg, for cognitive training or videos), which allows users to view content on another device via a QR code or by emailing it to themselves, was not considered intuitive to understand. The given tasks were missing additional information and were not clear without further explanation. Suggestions from the user testing included adding a notification for remembering to eat vegetables and fruits, as well as the marking of special input data values.

#### Functionalities of the LETHE App

##### Overview

**Figure 3:**
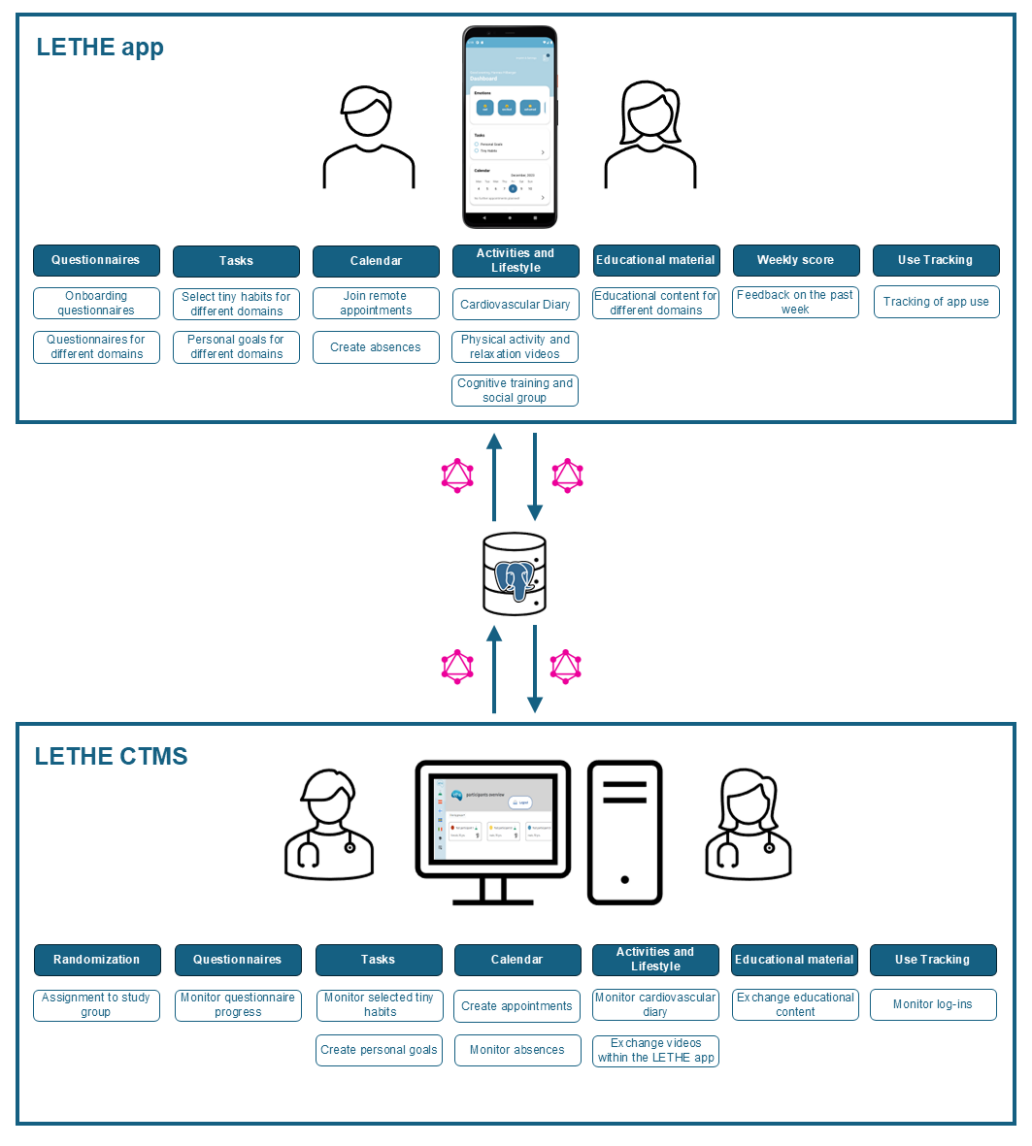
Overview of the modules, functionalities, and interactions between the LETHE app and the LETHE clinical trial management system (CTMS).

The trial incorporates the LETHE app, an Android-native app using Java, to facilitate digital aspects and streamline data collection from study participants. The LETHE app has been released through the Google Play Store and is publicly available for download. However, access to the app’s content is restricted to authorized study participants. Data storage and retrieval are managed through a .NET 7 (Core; .NET Foundation) application programming interface, which exposes data via GraphQL (Meta Platforms) protocols for external interaction with a PostgreSQL database (PostgreSQL Global Development Group). Figure 3 illustrates the modules of the LETHE app and the interaction with the LETHE CTMS.

**Figure 4:**
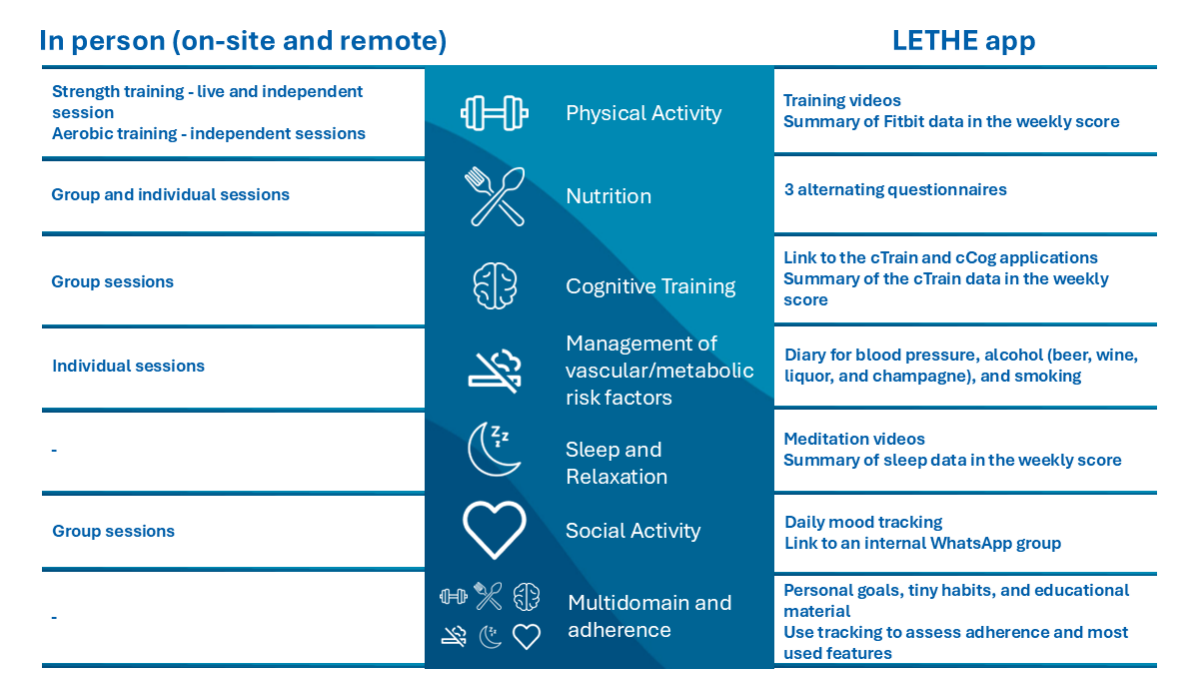
Comparison between in-person and digital components for each lifestyle domain.

The LETHE app correspondingly aligned the lifestyle domains with a specific digital feature based on the FINGER study [5] and included the additional domains of *sleep/relaxation* and *social activity*. Figure 4 illustrates a comparison between the in-person and digital components of the lifestyle intervention trial.

After completing the baseline study visit and initial questionnaires through the LETHE app, participants are randomly assigned to either the intervention or control group through the LETHE CTMS. Subsequently, the LETHE app dynamically adjusts the content based on group assignment. Both groups have access to the calendar, settings, questionnaires, mood tracking, and educational content. However, the intervention group has additional functionalities, such as personalized activities and the LETHE lifestyle program.

The LETHE app supports 5 languages (English, Finnish, Italian, Swedish, and German), with translations provided by the study centers. To enhance flexibility for features such as tiny habits and questionnaires, most of the translated content is dynamically retrieved, facilitating faster translation adjustments and content extensions. The translation process involved preparing an initial draft in English, which was then translated by native speakers at the clinical centers. The translations were reviewed to ensure accuracy for each region.

Designed with an older population in mind, the LETHE app uses a tile-based approach with large tiles as entry points for each functionality. This design allows individual elements to be hidden, shown, or reused, creating a modular structure. Following this approach, each component could be packaged independently, facilitating potential reuse in other research projects with similar requirements. Screenshots of each functionality of the LETHE app can be found in Multimedia Appendix 3.

##### Questionnaires

The questionnaires may be completed at single or multiple time points, with variations based on study group or participant gender. Each questionnaire consists of a title, due date, progress bar, and background color divided into green, yellow, and red, indicating the time remaining to complete the questionnaire. Participants can answer questions across multiple sessions, with answers cached on the device and sent only upon completion. The technical structure adheres to the Fast Healthcare Interoperability Resources (FHIR) standard [63], ensuring interoperability with other systems and enabling the questionnaire module’s reuse by incorporating different FHIR questionnaires while maintaining the same structure.

##### LETHE App Dashboard

Once assigned to either the intervention or control group, participants unlock access to the LETHE app dashboard, which consists of different tiles to access various functionalities. Participants can choose here how they feel daily. Each possible mood item is accompanied by a short description and an emoticon for visualization. The list of the available mood items is dynamically adaptable and could expand in future development based on participant feedback.

##### Tasks

The *Tasks* section encompasses the personalized intervention module, comprising 2 distinct areas: personal goals and tiny habits. Personal goals are collaboratively established with clinical professionals during visits, targeting specific lifestyle domains for personalized interventions. Personal goals follow the specific, measurable, achievable, realistic, and time-bound principle [64]. Unlike tiny habits, personal goals are not predefined, but they can be packaged as a future library of personalized objectives. These goals can be set daily, weekly, or monthly, offering frequency flexibility, including options such as bidaily or biweekly. Study participants can mark goals as completed or incomplete. Each personal goal is bound to a lifestyle domain, providing participants with an indication of their personal domains for lifestyle improvement.

Tiny habits provide practical everyday tips and behavioral suggestions to help individuals implement manageable healthy habits [65,66] and is adapted from the StopDia library [34], which is available in Finnish under a CC BY 4.0 license [67]. Tiny habits can be individually set by participants and are available for all lifestyle domains. Each tiny habit includes a description of the activity, a health fact about its benefits, and a place where it should be performed. There are >500 tiny habits in total to choose from, and they are assigned to a lifestyle domain. Given the subjective nature of tiny habit completion, participants are asked weekly about their success based on perceived completion in a questionnaire.

##### Calendar

The calendar displays all study-related appointments, both remote and in person, with a monthly and daily view. Remote visits can be joined directly from the daily view, which features larger tiles for individual appointments tailored to the target group. Participants can add vacation or unavailability periods, marking them as absences in the calendar. The absence menu is distinct in contrast to all other functionalities, providing a holiday like feel for participants.

##### Activity and Lifestyle

This section encapsulates the comprehensive LETHE app lifestyle program, featuring various activities tailored to distinct lifestyle domains. In the “Diary” section, study participants can log different health metrics such as blood pressure, alcohol consumption, and daily cigarette intake, directly contributing to the “Management of vascular/metabolic risk factors” lifestyle domain.

A range of activities aligned with different lifestyle areas unfold in the horizontal list at the top of the screen. It starts with a variety of videos dedicated to the areas of “Physical Activity” and “Relaxation.” For the “Cognitive Training” domain, there is a link to an external application called cTrain, a cognitive training game package encompassing games similar to those used in the original FINGER study [5,68].

For the “Social Activity” domain, participants from the intervention group can engage with a country-specific and internal WhatsApp group facilitated by clinical staff, ensuring exclusivity for trial participants. The last tile in the list serves as an educational hub, presenting web resources including web feeds provided by professionals via the LETHE CTMS for each lifestyle domain.

##### Weekly Scores

The weekly score with motivational feedback messages offers participants feedback on the previous week. The feedback message adjusts if the score increased or decreased. The score displays 3 rings representing “App Data/Lifestyle,” “Fitness,” and “Brain Training.” The “App Data/Lifestyle” category includes how often the LETHE app is used. The “Fitness” section displays Fitbit data, including step count, sleep duration, and individual fitness sessions, whereas “Brain Training” presents an overview of cognitive training data.

##### Use Tracking

To obtain insight into participants’ adherence and identify features of particular interest to older individuals in a lifestyle intervention app, a mechanism for use tracking was developed. Each time a participant opens the LETHE app, a unique ID is assigned for the session. Every screen is assigned to an event, and each event is saved with new time stamps when navigating through screens until the participant closes the app. This monitoring technique aims to understand useful features and incorporate measures for adherence analysis [69].

#### Evaluation of the LETHE App

##### Overview

Overall, of the 156 participants, 123 (78.8%) provided responses. The distribution of SUS scores in both the intervention group (*P*=.03) and the control group (*P*=.02) deviated significantly from normality. However, there was no significant difference in SUS scores between the groups (*P*=.18).

##### Intervention Group

**Figure 5:**
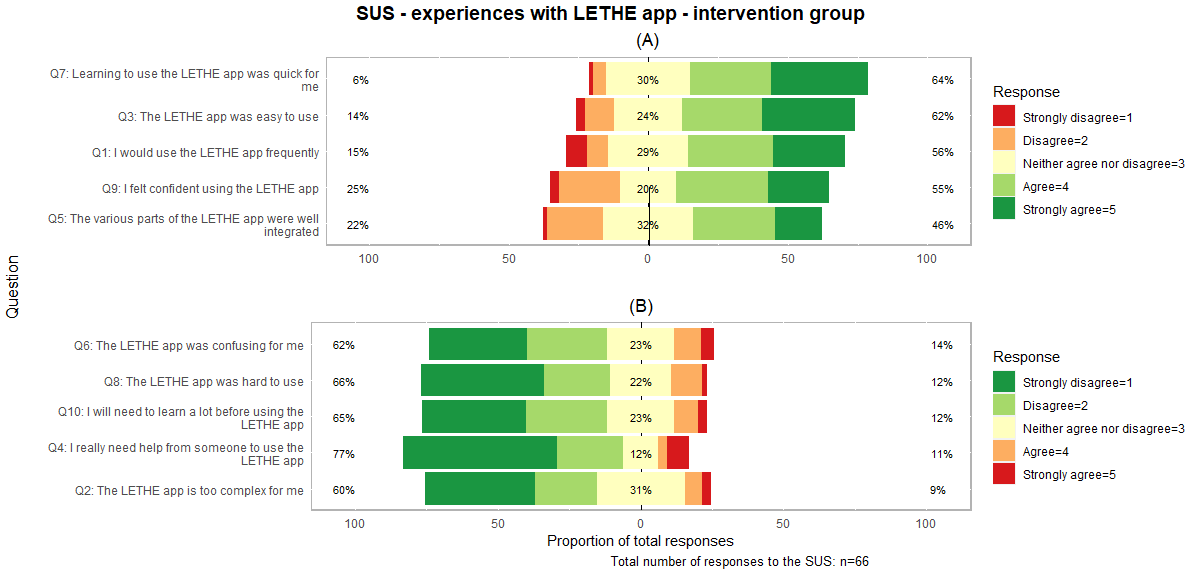
System Usability Scale (SUS) responses for the LETHE app in the intervention group. Panel (A) shows positively formulated questions, whereas panel (B) highlights negatively formulated questions. Green indicates high user satisfaction, and red indicates low user satisfaction. Q: question.

A total of 78 participants from the intervention group were invited for the SUS, and 66 (85%) responses were received. Of these 66 participants, 12 (18%) did not answer all the questions. The analysis revealed that the highest satisfaction levels were associated with learning how to use the app, whereas the lowest satisfaction levels were linked to the integration of various components, as shown in Figure 5 and Multimedia Appendix 4. The integration of the various components also received the most neutral responses among the questions. The question regarding the need for assistance when using the app generated the most disagreement, whereas learning about the app before use had the smallest response rate of 85% (56/66) for all questions.

Multimedia Appendix 4 provides an additional analysis for all 4 countries. Finland achieved the highest response rate (19/20, 95% of participant responses), and Sweden achieved the lowest response rate (12/18, 67%).

**Figure 6:**
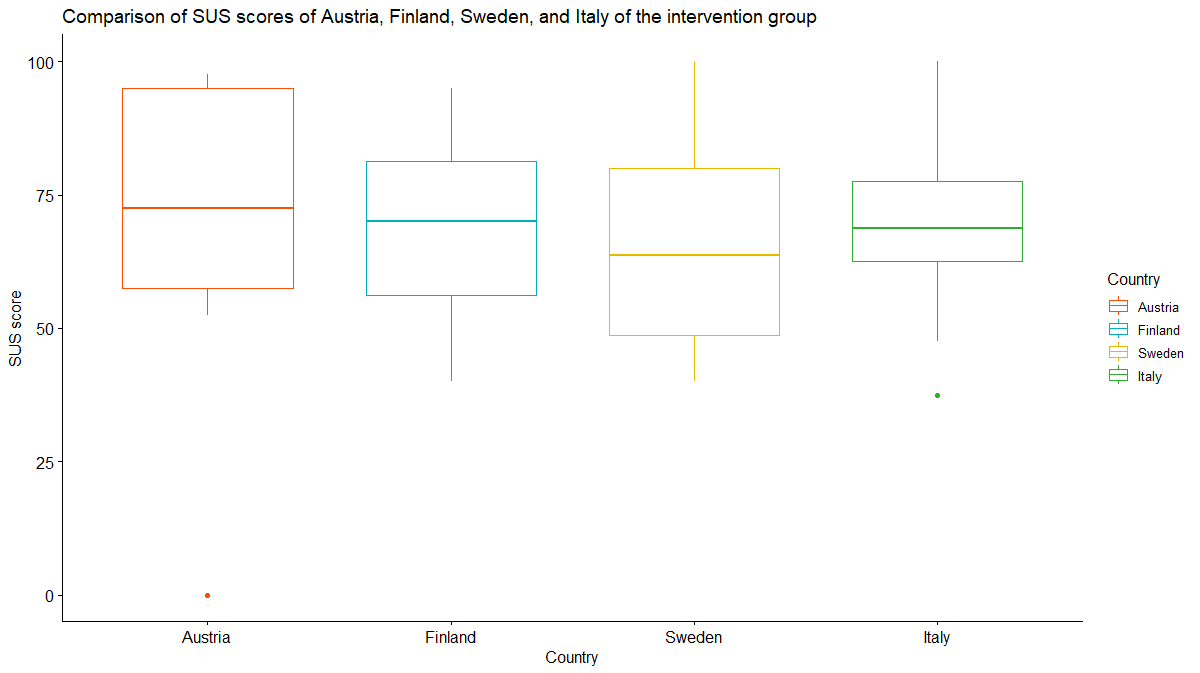
Box plot of the System Usability Scale (SUS) scores for each country in the intervention group.

As 18% (12/66) of the participants did not provide complete answers, missing values were replaced with the neutral value of 3 following the approach outlined by Lewis [70] to calculate the SUS. Figure 6 highlights the presence of 1 outlier with a total score of 0, which was not excluded.

The median SUS score was 70 (IQR 55-82), which is above the average score and qualifies the app for a grade C, as shown in Table 2.

**Table 2:** Scores on the System Usability Scale (SUS) and its subscales for study participants in the intervention group.

	Measure^a^	Values, median (IQR)	Grade
Total (n=66)	SUS	70 (55-82)	C
Austria (n=17)	SUS	73 (58-95)	C+
Finland (n=19)	SUS	70 (57-82)	C
Sweden (n=12)	SUS	64 (49-80)	C–
Italy (n=18)	SUS	69 (63-78)	C

^a^The SUS ranges from 0 to 100, with 100 as the highest score.

Austria achieved the highest median SUS score at 73 (IQR 58-95), whereas Sweden recorded the lowest median score at 64 (IQR 49-80). Austria was the only country where the app received a C+ score, distinguishing it from the others. Austria had 1 participant who was classified as an outlier with a total score of 0 but was still included in the analysis.

The distribution of SUS scores in Austria departed significantly from normality (*P*=.01). The distribution of SUS scores in Finland (*P*=.66), Sweden (*P*=.19), and Italy (*P*=.94) did not show evidence of nonnormality. The median SUS score did not differ significantly between the countries in the intervention group (*P*=.74).

##### Control Group

**Figure 7:**
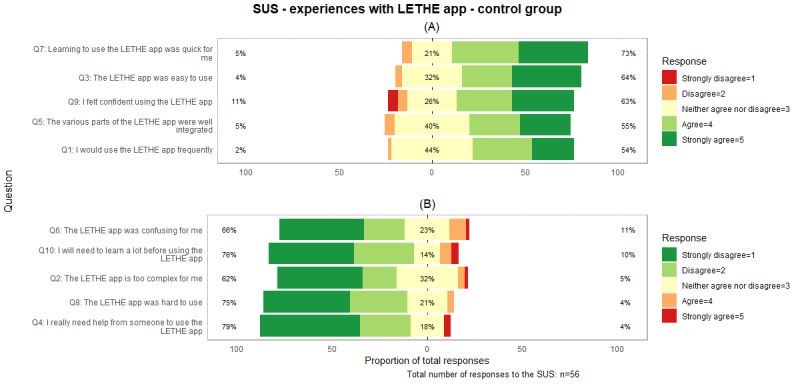
System Usability Scale (SUS) responses for the LETHE app in the control group. Panel (A) shows positively formulated questions, whereas panel (B) highlights negatively formulated questions. Green indicates high user satisfaction, and red indicates low user satisfaction. Q: question.

For the limited version of the LETHE app, 73% (57/78) of the study control group participants responded. Notably, 18% (10/57) of the respondents did not answer all questions. Figure 7 and Multimedia Appendix 5 indicate that the highest satisfaction was achieved in terms of how quickly participants could learn to use the LETHE app, followed by its ease of use. The only instance of a “Strongly disagree” response was regarding the confidence in using the LETHE app. The question about the LETHE app’s frequent use gathered the most neutral responses, possibly due to its limited functionalities compared to the intervention version. A total of 79% (44/56) of the participants disagreed with the idea of needing help from others while using the LETHE app. Similarly to the intervention group, the question about learning about the LETHE app before using it received the smallest response rate of 98% (56/57) of all questions.

In the control group, Italy exhibited the lowest response rate, with 60% (12/20) of the participants responding, whereas Austria had the highest response rate, with 85% (17/20) of the participants responding. A detailed breakdown of responses per country is available in Multimedia Appendix 5.

To calculate the SUS, all missing values were substituted with the neutral value of 3, mirroring the approach taken with the intervention group. Table 3 illustrates that the median SUS score was 73 (IQR 63-90), corresponding to a B– grade.

**Table 3:** Scores on the System Usability Scale (SUS) and its subscales for study participants in the control group.

	Measure^a^	Values, median (IQR)	Grade
Total (n=57)	SUS	73 (63-90)	B–
Austria (n=17)	SUS	90 (73-95)	A+
Finland (n=16)	SUS	69 (65-76)	C
Sweden (n=12)	SUS	64 (50-84)	C–
Italy (n=12)	SUS	78 (54-90)	B+

^a^The SUS ranges from 0 to 100, with 100 as the highest score.

**Figure 8:**
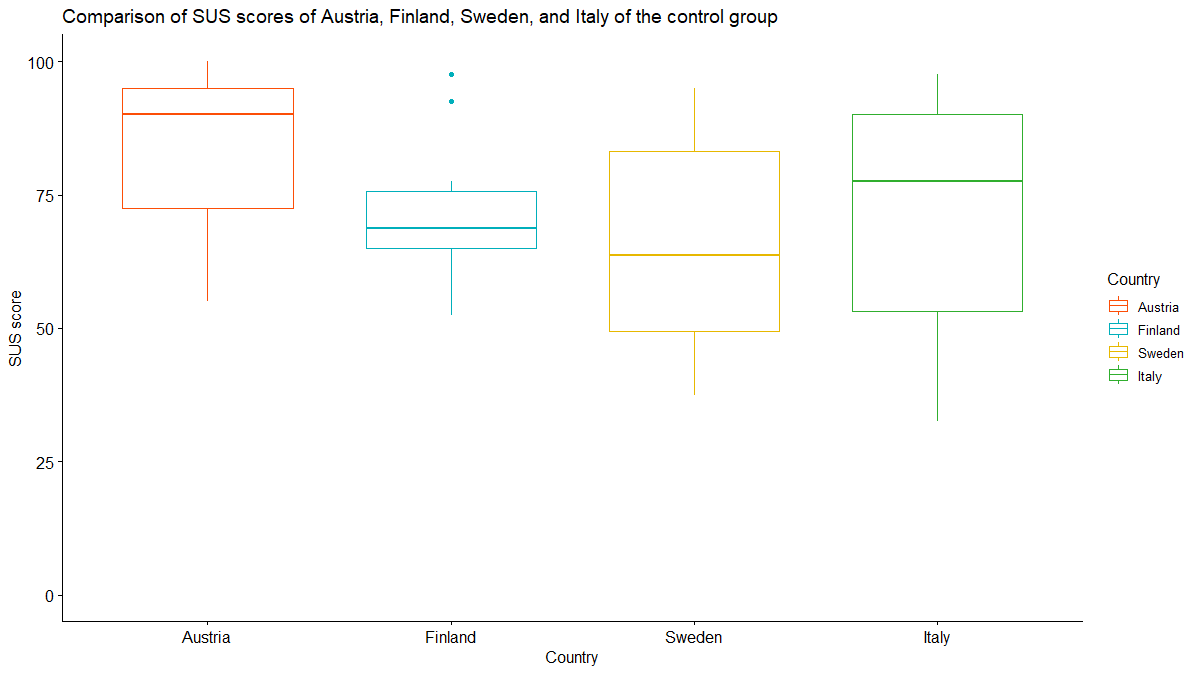
Box plot of the System Usability Scale (SUS) scores for each country in the control group.

Austria had the highest median SUS score at 90 (IQR 73-95), giving the app an A+ grade, whereas Sweden had the lowest median score at 64 (IQR 50-84), giving the app a C– grade. The app obtained a C grade in Finland and a B+ grade in Italy, with median scores of 69 (IQR 65-76) and 78 (IQR 54-90), respectively. Notably, in contrast to the intervention group, the median SUS score was higher in Austria and Italy and lower in Finland, and it stayed the same in Sweden. Figure 8 visualizes the box plot for each country.

The SUS scores of Austria (*P*=.03) and Finland (*P*=.047) departed significantly for normality. The distribution of the SUS scores of Sweden (*P*=.41) and Italy (*P*=.11) did not show evidence of nonnormality. The SUS scores did differ between the countries in the control group (*P*=.03). Pairwise comparisons showed that the median SUS score of Austria was significantly different than the median SUS score of Sweden (*P*=.02). For the other pairwise comparisons, no significant difference was found. Specifically, the comparisons between Austria and Finland (*P*=.33), Austria and Italy (*P*=.36), Finland and Sweden (*P*=.99), Finland and Italy (*P*=.99), and Sweden and Italy (*P*=.99) all yielded *P* values greater than the threshold for statistical significance.

### Design, Functionalities, and Evaluation of the LETHE CTMS

#### Feedback From the Workshop

After an examination of the LETHE study protocol and the examples from the literature [55-57] for a design perspective, initial mock-ups for the LETHE CTMS, a web application, were created. The mock-ups were kept very general and built the foundation for the discussions in the workshop with health professionals. These mock-ups encompassed (1) an overview page featuring details on all study participants, (2) a dedicated view for an individual study participant, (3) the conceptualization of an AI simulation, and (4) a data entry page aligned with the study protocol.

The feedback on the mock-ups included the exclusion of real names given that all study participants would be collectively visible on one page in the mock-up. Emphasis was placed on the immediate visibility of adherence using colors (eg, green, yellow, and red) and dropouts. In addition, diverse roles were implemented to restrict access to sensitive information, such as ensuring that health professionals could only view data related to their own country as well as a blinded role to not see the group of the study participants. Workshop participants also expressed the desire for a descriptive overview of the dataset variables.

Subsequently, the discussion turned to the overview page, where all study participants are listed. Key considerations included the inclusion of adherence information and the need for clear differentiation between participants from the intervention and control groups. Given the sizable participant list of 156 individuals, there was a request for sorting and search functionalities.

During discussions regarding the page presenting information for an individual study participant, workshop participants advocated for distinct perspectives tailored to individuals in the intervention and control groups, emphasizing adherence and dropout summaries. Further dialogues included role-specific views, aiming for the visibility of only relevant data. The workshop participants also expressed a preference for a comprehensive presentation of lifestyle categories, including detailed information and distinct sections for different clinical professionals as well as additional risk factors such as the consumption of alcohol or cigarettes. Requests for additional functionalities included the presentation of the Clinical Dementia Rating [71], allergies, questionnaire responses and scores, notes on participant interactions, details of adverse events such as muscle pain after a prescribed aerobic workout, and automated calculations such as BMI. Another necessity identified was the inclusion of a data export functionality.

When considering the AI dementia risk simulation, there was a discussion about whether it could be used during the study intervention or solely for research purposes. The AI risk simulation is currently available only to researchers due to ethical considerations. As the models used in the simulation have not yet been fully tested or validated, they were not implemented with real participants at this stage. Moreover, the risk information generated by the simulation is not disclosed to participants. Should the disclosure of this risk information be considered in the future, it would require careful evaluation. This process would involve consulting with both the participants and experts in ethics to assess the potential psychological impacts and the broader ethical implications of sharing risk-related information with participants.

The workshop discussed the insertion of all CRF data into the LETHE CTMS, encompassing visit-related data and scores such as the Clinical Dementia Rating and Neuropsychological Test Battery as in the FINGER study. Workshop participants underscored the inclusion of medications, validation checks, and prefillable fields. The study protocol served as a reference for defining the data entry page fields.

Following the conclusion of the workshop, participants were given a clickable prototype. Subsequently, a follow-up workshop was conducted to incorporate adaptations based on the initial workshop insights. The most significant modification was made to the data entry page, necessitating a complete restructuring due to a higher-than-anticipated number of data fields. After several iterations, a design for the beginning of the study was proposed.

#### Functionalities of the LETHE CTMS

##### Overview

Following the completion of the LETHE CTMS design, pivotal components were identified to commence the clinical trial. This included an overview of all study participants, a detailed view of a study participant, and the data input portal for clinical professionals. The LETHE CTMS was developed as a web application using React and uses the same backend infrastructure as the LETHE app. The LETHE CTMS is deployed as a Docker container (Docker, Inc). Once a new version is pushed to the main branch of the GitLab repository, a Continuous Integration/Continuous Delivery or Deployment (CI/CD) pipeline is automatically triggered, handling the build, release, and deployment process. After the container is pushed to the GitLab container registry, deployment is carried out using an Ansible playbook (Ansible Inc).

The LETHE CTMS has a consistent design throughout the application, achieved by using a fundamental page structure featuring headers, card-based content representation, a uniform color scheme inspired by the LETHE project colors, and font selection. Ensuring that the data are relevant only to the relevant users was achieved through ongoing communication with end users, and various roles were defined. There are roles that have access to all participants from all study centers; roles that are blinded and, therefore, can only enter visit forms; and roles that are not blinded and can have access to all participant details from their study center.

The LETHE CTMS underwent iterative modifications over time driven by continuous user feedback and evolving requirements. In particular, each section has been given a standardized nomenclature. At the moment, the following sections are included: overview page, detail page, clinician data entry page (containing the electronic CRF [eCRF]), and the configuration pages to adapt the content in the LETHE app. For now, the AI risk simulation is only available for research purposes and not visible to the end users, so a further description is not provided. Screenshots of the different pages can be found in Multimedia Appendix 6.

##### Overview Page

The overview page serves as a visual representation of all study participants within the selected country. Upon initializing this page, the study participants are loaded based on the study site affiliation of the clinical professional. The header features a legend outlining variables encompassing distinct adherence pathways and the respective participant groups. Color-coded adherence pathways range from “low” (red) to “medium” (yellow) to “high” (green) and include a category of “not calculated.” These pathways are grouped into distinct types, with more details provided on the detail page.

The central section of the page displays all participants from the current site within individual rounded boxes, which encompasses variables such as the participant’s ID, and other essential participant information, including adherence, group affiliation, age, and gender, is also presented.

There is also the possibility to access the configuration pages, allowing for the modification of content related to educational material and videos on the LETHE app.

##### Detail Page

Upon selecting an individual on the overview page, a more detailed view is presented, encompassing comprehensive data on that particular participant.

The detailed view interface is organized into 3 columns, each with functional components. In the first column, baseline visit data are featured, encompassing demographics, cardiovascular metrics, comorbidities, medications, allergies, and blood values. Users can review completed and pending questionnaires as well as access the responses and corresponding scores. This column concludes with a messaging feature that allows professionals to schedule notifications directly sent to study participants’ smartphones.

The second column commences with notes about adverse events, the entering and tracking of personal goals, and the monitoring of the selected tiny habits and cardiovascular risk factor data entered into the LETHE app. Professionals can also view participant absences to adjust appointment scheduling and reschedule personal goals accordingly.

In the last column, professionals can schedule recurring remote or in-person meetings with study participants together with reminders. Each meeting can be marked as completed to help track the adherence. Participant-professional contacts are recorded, and additional information is displayed, including last LETHE app log-in, participant consent status, and participation in substudies.

At the bottom of the page, adherence to various categories, such as app use, cognitive activity, diet, and physical activity, is visually displayed, providing an overview of the participant’s engagement with key aspects of the study protocol. Table 4 shows the thresholds used to assign each pathway to one of the adherence levels. On the basis of adherence and lifestyle domain, participants receive tailored messages on specific weekdays: physical activity on Mondays, app use on Tuesdays, diet on Wednesdays, cognitive activity on Thursdays, cardiovascular risk factors and social interaction on Fridays, and relaxation and sleep on Saturdays. Participants consistently in the red pathway receive domain-specific or holistic messages. In addition, tiny habit messages are sent on Mondays, Wednesdays, and Thursdays based on reported engagement.

**Table 4:** Adherence pathways and thresholds for different categories.

	Data type	Instrument used to measure the data and frequency of measurement	Green threshold	Yellow threshold	Red threshold
Physical activity	Activity min and frequency (moderate to vigorous intensity)	Fitbit; weekly (Mondays); passive	Months 1-3: 30-45 min (1 time per wk); months 4-6: 30-45 min (2-3 times per wk); months 7-9: 30-60 min (3-4 times per wk); months 10-24: 45-60 min (3-5 times per wk)	Months 1-3: 15-29 min (1 time per wk); months 4-6: 15-29 min (1 time per wk); months 7-9: 15-29 min (1-2 times per wk); months 10-24: 15-44 min (1-2 times per wk)	1 wk with 0 min or 3 weeks in a row in the yellow path
App use	Changes between the screens within the LETHE app	App; weekly (Tuesdays); passive	≥3 screens (4-7 d)	≥3 screens (0-3 d)	0 screens for 14 d
Diet patterns	Completing food-monitoring item questionnaire within the LETHE app (3 alternating questionnaires)	App; weekly (Wednesdays); active	2/3 or 3/3 blocks completed (in 3 wks)	1/3 blocks completed (in 3 wks)	0% completed (in 3 wks)
Cognitive training	Use of cognitive training program	cTrain; weekly (Thursdays); active	2-3 times per wk	0-1 times per wk	0 times for 3 wk

##### Data Entry Page

Diverging from the workshop, a series of design iterations led to the decision that the data entry page would act as a repository for information gathered from all study visits, whereas tasks such as contacting study participants and scheduling appointments were moved to the detailed view of an individual participant.

Within the eCRF, each primary section, covering screening, baseline, and subsequent visits, contains subforms that address different documentation areas (eg, forms to report results for blood tests, medication use, and neurological assessments such as the Mini-Mental State Examination [72]).

Each subform within the eCRF incorporates features such as automatic calculations and validation checks, whereby abnormal values trigger a visual highlighted in yellow. This ensures data accuracy and enhances the efficiency of data entry. The eCRF design, embedded with these functionalities, serves as a comprehensive and streamlined platform for recording and managing diverse aspects of study visit documentation.

#### Evaluation of the LETHE CTMS

##### Overview

This section analyzes the feedback from the CTMS survey completed by health professionals. First, user-specific details, such as their role in the project and within the application are presented. Second, the quantitative and qualitative feedback for each of the functionalities and the design is analyzed. Finally, a ranking of the state-of-the-art features is presented. The survey refers to the LETHE CTMS as *LETHE dashboard*, but both terms are interchangeable.

##### Users of the LETHE CTMS

The survey targeted all users of the LETHE CTMS, gathering general information such as their role in the project, their function within the application, and the frequency of their application access. When the survey was conducted, 21 users had access to the LETHE CTMS, and 15 (71%) responded to the survey. The summarized results are presented in Table 5. Most respondents held roles as coordinators or in unblinded positions, with access to almost all features. Of the 15 respondents, 12 (80%) accessed it at least once a week.

**Table 5:** Role of professionals in the LETHE project and in the LETHE clinical trial management system (CTMS) and frequency of CTMS use (N=15).

Answer option		Professionals, n (%)
“**Could you please describe your role in the project?”**		
	Coordinator	1 (7)
	Digital coach	2 (13)
	Neuropsychologist	3 (20)
	Nutritionist	2 (13)
	Physiotherapist	1 (7)
	Principal investigator	1 (7)
	Study nurse	4 (27)
	Study physician	1 (7)
“**What dashboard role do you have?”**		
	Blinded role	1 (7)
	Coordinator role	6 (40)
	Unblinded role	8 (53)
“**How frequently do you access the dashboard?”**		
	Daily	1 (7)
	More than once a week	6 (40)
	Once a week	5 (33)
	More than once a month	1 (7)
	Once a month	2 (13)

##### Feedback on the Design and Functionalities of the LETHE CTMS

**Figure 9:**
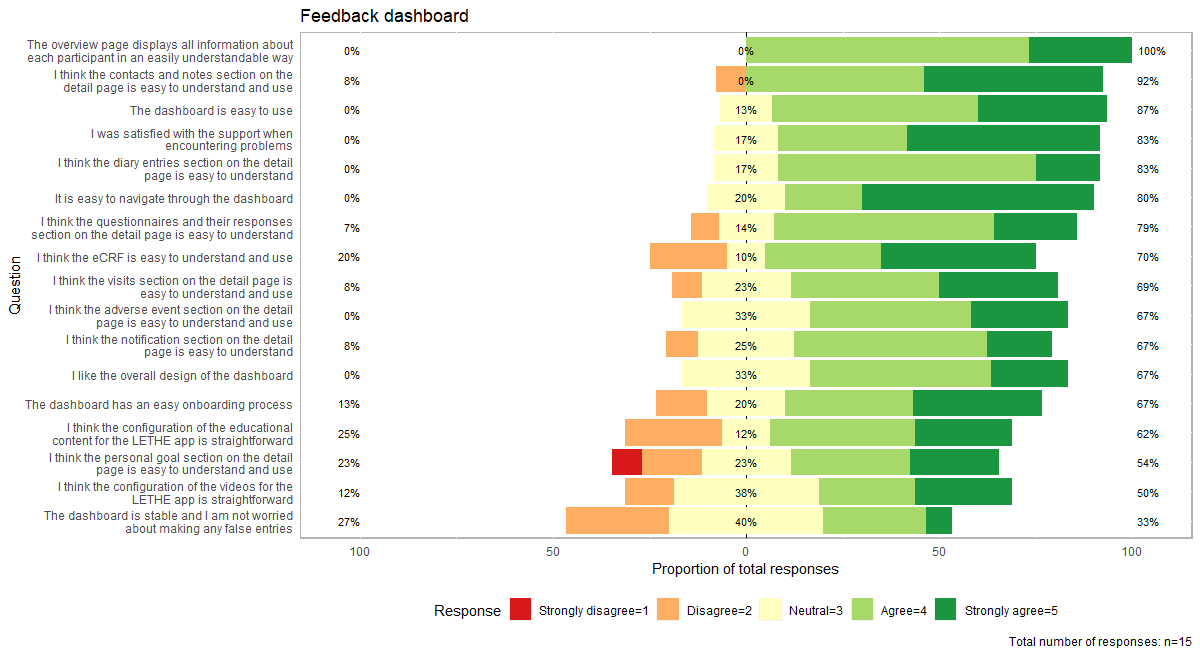
Survey responses for the LETHE clinical trial management system from health professionals. Green indicates high user satisfaction, and red indicates low user satisfaction.

Figure 9 and Table 6 indicate that the highest level of user satisfaction was related to the comprehensive display of relevant participant information in an easily understandable way, with a score of 100% above neutral. A detailed breakdown of all quantitative responses can be found in Multimedia Appendix 7. Overall, feedback was positive, reflecting successful integration of design approaches from workshops and feedback. Users found the LETHE CTMS intuitive, with a visually appealing and clear layout featuring consistent colors, logically divided sections, and pictograms. Pop-ups during item saving aided navigation and provided feedback on actions. Suggested improvements included reducing the steps to move between participants, addressing inconsistencies in single- and double-click requirements, and ensuring visibility of study participant phone information on the participant overview page.

**Table 6:** Means and SDs of the LETHE clinical trial management system feedback survey targeting the different functionalities.

	Values, mean (SD)^a^
“The overview page displays all relevant information about each participant in an easily understandable way” (n=15)	4.27 (0.46)
“I think the Contacts/Notes Section on the Detail Page is easy to understand use” (n=13)	4.31 (0.85)
“The Dashboard is easy to use” (n=15)	4.20 (0.68)
“I was satisfied with the support when encountering problems” (n=12)	4.33 (0.78)
“I think the Diary Entries Section on the Detail Page is easy to understand” (n=12)	4.00 (0.60)
“It is easy to navigate through the Dashboard” (n=15)	4.40 (0.83)
“I think the Questionnaires and their Responses Section on the Detail Page is easy to understand” (n=14)	3.93 (0.83)
“I think the eCRF is easy to understand and use” (n=10)	3.90 (1.20)
“I think the Visits Section on the Detail Page is easy to understand and use” (n=13)	3.92 (0.95)
“I think the Adverse Event Section on the Detail Page is easy to understand and use” (n=12)	3.92 (0.79)
“The Dashboard has an easy onboarding process” (n=15)	3.87 (1.06)
“I like the overall design of the Dashboard” (n=15)	3.87 (0.74)
“I think the Notification Section on the Detail Page is easy to understand” (n=12)	3.75 (0.87)
“I think the configuration of the Educational Content for the LETHE App is straightforward” (n=8)	3.63 (1.19)
“I think the Personal Goal Section on the Detail Page is easy to understand and use” (n=13)	3.46 (1.27)
“I think the configuration of the Videos for the LETHE App is straightforward” (n=8)	3.63 (1.06)
“The Dashboard is stable and I am not worried about making any false entries” (n=15)	3.13 (0.92)

^a^The scale ranges from 1 (*Strongly disagree*) to 5 (*Strongly agree*). A higher level of agreement indicates greater satisfaction.

Concerning the onboarding process, users expressed that it involved multiple steps and should be streamlined further. However, once granted access, the general opinion was that the process was straightforward. The lowest satisfaction regarding functionalities was observed in the educational content and video configuration, where users missed options for uploading videos and materials instead of web links. The stability and prevention of false entries, particularly in the eCRF, received the lowest satisfaction rating. Users reported issues with scrolling affecting eCRF values and occasional problems with saving values. Suggestions included implementing a log to track data edits for added transparency, a measure already in place but not visible to users. Another notable disagreement in the *Personal Goals* section was that users found it difficult to set goals at regular intervals, and the lifestyle domain associated with each personal goals was not visible in the detailed view of a study participant.

Feature suggestions included displaying follow-up data, editing values in different sections, providing additional Fitbit data insights, and summarizing clinical test names and scores at the bottom of the eCRF page. Positive aspects highlighted satisfaction with support, emphasizing quick response times, correction of implementations, and clear explanations.

Other feedback suggested the ability to send messages to groups of study participants as well as create group meetings. Overall, those surveyed commented on the effectiveness of the LETHE CTMS for study staff and participants, noting a continuous improvement since its first use.

##### Ranking the Most Useful and Beyond-State-of-the-Art Features

One of the survey questions asked respondents to rank various features integrated into the LETHE CTMS based on their perceived usefulness and how they surpassed state-of-the-art capabilities. As shown in Table 7, the integration of personalized activities (mean 2.23, SD 1.17) and real-time appointment planning (mean 2.46, SD 1.51) stood out as the most impactful features. An additional comment from a respondent highlighted the novelty of the interplay between the LETHE CTMS and the LETHE app, with a note that further optimization was needed. In addition, the LETHE CTMS holds the potential to serve as both an eCRF and a tool to assist in intervention delivery. This dual functionality was seen as a valuable development for the future to meet the evolving needs of professionals conducting clinical trials.

**Table 7:** Ranking of different features based on surpassing state-of-the-art capabilities, with R1 being the best-ranked feature and R7 being the worst-ranked feature.

	Choices (n=13), n (%)								Total (n=91), n (%)
	C1^a^	C2^b^	C3^c^	C4^d^	C5^e^	C6^f^	C7^g^		

R1	2 (15)	2 (15)	1 (8)	0 (0)	1 (8)	4 (31)	3 (23)	13 (14)	
R2	1 (8)	0 (0)	0 (0)	0 (0)	1 (8)	4 (31)	7 (54)	13 (14)	
R3	1 (8)	0 (0)	2 (15)	4 (31)	3 (23)	2 (15)	1 (8)	13 (14)	
R4	0 (0)	3 (23)	3 (23)	3 (23)	1 (8)	2 (15)	1 (8)	13 (14)	
R5	1 (8)	1 (8)	3 (23)	2 (15)	5 (38)	0 (0)	1 (8)	13 (14)	
R6	2 (15)	5 (38)	1 (8)	3 (23)	1 (8)	1 (8)	0 (0)	13 (14)	
R7	6 (46)	2 (15)	3 (23)	1 (8)	1 (8)	0 (0)	0 (0)	13 (14)	
Total (n=91)	13 (14)	13 (14)	13 (14)	13 (14)	13 (14)	13 (14)	13 (14)	91 (100)	

^a^Overview page of different study participants in different countries, including their digital intervention adherence pathways (mean 5.08, SD 2.43).

^b^Direct notifications to study participants via the dashboard (mean 4.84, SD 1.99).

^c^Electronic case report form (mean 4.69, SD 1.80).

^d^Immediate results of onboarding and questionnaires on the dashboard (mean 4.54, SD 1.40).

^e^Configuration of the content of the app via the dashboard (mean 4.15, SD 1.68).

^f^Real-time planning of appointments within the dashboard for the app (mean 2.46, SD 1.51).

^g^Integration of personalized intervention tasks (*Personal Goals*; mean 2.23, SD 1.17).

## Discussion

### Principal Findings

This paper outlines the design process, functionalities, and evaluation of the digital intervention study components within the LETHE project, focusing on older individuals at risk of cognitive decline and clinical professionals. The design process involved multiple sessions with potential end users and clinical experts. Furthermore, the setup is currently being evaluated in a 2-year intervention study, and the first results have been presented [11,69].

The original FINGER multidomain trial, along with related studies, demonstrated that cognitive benefits can be achieved through a 2-year intervention. As a result, this duration was established as the timeline for the LETHE trial. However, there is only limited information available on longer studies supported by ICT components, such as digital apps requiring daily interactions and wearable devices.

These requirements made it necessary to carefully design the study in terms of human support and create user-friendly ICT components for both participants and clinical professionals. The learnings regarding ICT use in clinical studies, as well as data collected in intervention studies (clinical data and digital biomarkers), are essential and can influence future studies and clinical trial setups using ICT.

To emphasize the importance of user-friendly design, this paper presents the process from requirement gathering to the design of the components. The findings will be evaluated and will inform further improvements to the setup.

Overall, the median SUS score of the intervention group of 70 (IQR 55-82) was comparable to that of the control group of 73 (IQR 63-90). The slightly higher median score of the control group might be attributed to the less complex functionalities compared to those of the intervention group version, specifically the absence of personalized and intervention activities. However, both median scores surpassed 68, indicating above-average user acceptance and satisfaction according to SUS guidelines [62]. Given that the initial user experience for the control group was quite positive even with fewer features, there is hope that they will remain engaged throughout the trial. It is anticipated that these values will improve as participants gain more experience with the LETHE app during the 2-year RCT [73]. Evaluating the other time points will give us more information about the longer-term usability and user engagement.

Noteworthy is the variation in median SUS scores between countries, with Austrian participants providing the highest scores (intervention group: median 73, IQR 58-95; control group: median 90, IQR 73-95) and Sweden providing the lowest scores (intervention group: median 64, IQR 49-80; control group: median 64, IQR 50-84). Performing a pairwise comparison using the Dunn test, a significant difference in median SUS scores between Austria and Sweden was found (*P*=.02).

While the LETHE lifestyle intervention program is centrally coordinated, and the activities in the 4 countries are harmonized to ensure comparable content, certain local adaptations are allowed to optimize feasibility (eg, in the detailed intervention delivery [balance between in-person and digital sessions] and how the digital components are leveraged). A qualitative study is currently underway to collect additional data on the barriers to and facilitators of the use of digital tools among participants in the LETHE trial.

Overall, users expressed satisfaction with the LETHE CTMS, and workshops on design, usability, and functionality proved beneficial. However, clarity regarding the purpose of an AI risk simulation and addressing user concerns about inaccurate data entry emerged as key challenges during the design process. Further exploration into user satisfaction with the intervention’s introduction by clinical professionals across different countries is warranted for future research.

Research should investigate reasons behind decreased personal engagement in long-term ICT-supported studies. The LETHE protocol, with its 2-year ICT-supported design, offers a unique contribution to support cognitive health in older individuals.

In terms of future research, several key directions should be explored. First, a thorough evaluation of the platform’s use and usability after the completion of the full 2-year trial would provide valuable insights into its long-term effectiveness. Second, further investigations could focus on the AI-driven risk simulation within the CTMS, with a particular emphasis on its potential role in guiding future interventions. Finally, exploring the adaptability of the LETHE platform among populations with low digital literacy and assessing the feasibility of a hybrid approach would be an important area for future research.

We are confident that LETHE app’s diverse features, together with high retention, positive feedback and frequent use [11,69], provide promising directions for future hybrid multimodal interventions.

### Strengths and Limitations

This study has several strengths, particularly in how the LETHE app and CTMS incorporate perspectives from both northern and southern Europe. This allows for a broader range of views to be considered. Moreover, this study is regularly improved through feedback from professionals, suggestions from study participants, and input from advisory board members, ensuring ongoing refinement and relevance. One limitation lies in assuming that all participants have sufficient digital skills and internet access, which was an inclusion criterion for the RCT. However, according to a recent study, the gap in digital health trends between younger and older people may vanish in 10 years as today’s individuals aged <65 years are highly adapted to digital solutions [74].

Furthermore, all participants were provided with introductory materials and manuals for the LETHE app to facilitate use. Those factors could impact their ratings. Moreover, the subjective nature of the SUS, which relies on participants’ self-reported perceptions of usability, might not fully grasp the nuanced usability challenges, especially among older users who may have varying cognitive and physical abilities. Another limitation is that the LETHE app content (eg, educational materials or videos) varies between countries and may influence the ratings.

Another consideration is that the initial user testing involved a small sample size, with only 4 German-speaking participants for the first user testing. However, the evaluation of the LETHE app is part of a study in which health professionals and 156 study participants are included.

The LETHE app was developed exclusively for the Android platform. This decision was based on practical considerations, including resource constraints, time limitations, and the need for a streamlined pilot phase. Focusing on a single operating system allowed for a more efficient development, testing, and release process, ensuring that the feasibility trial could be completed within the available time frame. Android was chosen due to its widespread use and compatibility with various devices, facilitating broader accessibility. To mitigate potential accessibility barriers, participants who did not have an Android device were given the option to receive one for the study. This approach enabled a standardized evaluation of the app’s functionality and user experience. Future iterations of the app may consider cross-platform availability, including an iOS or web version.

Regarding the LETHE CTMS, a limitation of the survey is that most users held roles with the highest level of privileges in terms of functionalities, with frequent role changes occurring throughout the project. Improved planning of roles is advisable for future applications to mitigate this issue.

### Comparison With Prior Work

Previous studies indicate declining user engagement with mHealth interventions over time due to multiple factors, such as unengaging content, complex protocols, or poor design. For instance, studies such as predictive model-based decision support for diabetes patient empowerment (POWER2DM) [75] have investigated ICT-supported health systems integrating features such as shared decision-making, personal goal setting, mood tracking, and exercise components, which is similar to the interaction between the LETHE app and LETHE CTMS but within the context of diabetes self-management.

Compared to existing digital interventions for dementia prevention, such as PRODEMOS [15] and Maintain Your Brain [16], LETHE introduces several additions and refinements. LETHE builds on standard goal setting but also includes the tiny habits method as an add-on. It also covers additional lifestyle domains, including social engagement and sleep, which are not included in PRODEMOS but are, meanwhile, part of the Lancet Commission’s report [2].

In terms of data collection, LETHE uses Fitbit devices to passively monitor physical activity and sleep, whereas PRODEMOS relies on self-reported step counts. Both interventions use a hybrid approach, but LETHE’s study duration is extended to 24 months (compared to 18 months in PRODEMOS) and builds on the successful FINGER protocol [5].

LETHE differs from Maintain Your Brain by offering immediate access to all modules rather than introducing them sequentially. Although LETHE does not include a separate mental health module, it collects related information through questionnaires. Maintain Your Brain, compared to LETHE, is entirely internet based and spans 3 years, with only quarterly booster activities after the first year and yearly follow-up assessments for the remaining 2 years.

Finally, unlike both PRODEMOS and Maintain Your Brain, a primary outcome of LETHE is the feasibility of a digitally supported multimodal lifestyle intervention, assessed through participant engagement with the LETHE app and a Fitbit device, attendance to study activities, and implementation of a hybrid approach that combines active and passive data collection across 4 countries.

One significant factor contributing to the unsatisfactory use of ICT components in ICT intervention studies is the usability of the functions provided. This paper provides a comprehensive account of the design process for both the app and the CTMS, aiming to maximize their usability. Subsequent publications will detail the outcomes concerning use and user satisfaction. Identified gaps in involving older adults in the design process of digital health technologies shed light on issues such as the exclusion of individuals with low digital skills during recruitment, emphasizing the importance of using diverse methods such as focus groups, interviews, or workshops [76]. While both the LETHE app and LETHE CTMS design processes incorporated various methods to solicit continuous feedback from diverse user populations, they did not explicitly include persons with low digital skills.

### Conclusions

In summary, this paper presented and evaluated the design and functionalities of a comprehensive clinical trial system involving a mobile app tailored for older individuals at risk of cognitive decline. The LETHE app and a web-based monitoring and configuration system for clinical professionals, the LETHE CTMS, were thoroughly evaluated through survey-based assessments. Overall, our approach facilitates real-time interaction, providing 2 distinct applications for professionals and study participants. The results elucidated the design principles, stakeholder involvement, and essential functionalities of such an eHealth system. Subsequent research will delve into the posttrial use of the LETHE app over the 2 years, shedding further light on its effectiveness and user engagement.

## Appendix

Multimedia Appendix 1 LETHE app survey.

Multimedia Appendix 2 LETHE clinical trial management system survey.

Multimedia Appendix 3 LETHE app screenshots.

Multimedia Appendix 4 LETHE app evaluation—intervention group.

Multimedia Appendix 5 LETHE app evaluation—control group.

Multimedia Appendix 6 LETHE clinical trial management system screenshots.

Multimedia Appendix 7 LETHE clinical trial management system cross-table.

## Abbreviations

AI: artificial intelligence
CI/CD: Continuous Integration/Continuous Delivery or Deployment
CRF: case report form
CTMS: clinical trial management system
eCRF: electronic case report form
FHIR: Fast Healthcare Interoperability Resources
FINGER: Finnish Geriatric Intervention Study to Prevent Cognitive Impairment and Disability
ICT: information and communications technology
mHealth: mobile health
POWER2DM: predictive model-based decision support for diabetes patient empowerment
PRODEMOS: prevention of dementia using mobile phone applications
RCT: randomized controlled trial
SUS: System Usability Scale
UCD: user-centered design
