# Supplementary material for: Design of a Mobile App and a Clinical Trial Management System for Cognitive Health and Dementia Risk Reduction: User-Centered Design Approach
Source: JMIR Aging. 2025 Jul 2;8:e66660. doi: 10.2196/66660 (PMC12268216; doi:10.2196/66660)
Supplement: Multimedia Appendix 1 [file aging_v8i1e66660_app1.pdf]

This is a Multimedia Appendix to a full manuscript published in the J Med Internet Res. For full copyright and citation information see http://dx.doi.org/10.2196/jmir.66660

English:
Experiences with LETHE app

In the following questionnaire, you will be asked questions about your experience with the LETHE app. Please read the questions and any related instructions carefully before answering. The questionnaire consists of 10 questions, which should take approximately 2 minutes to answer.

	Strongly disagree - 1	2	3	4	Strongly agree - 5
1. I would use the LETHE app frequently					
2. The LETHE app is too complex for me					
3. The LETHE app was easy to use					
4. I really need help from someone to use the LETHE app					
5. The various parts of the LETHE app were well integrated					
6. The LETHE app was confusing for me					
7. Learning to use the LETHE app was quick for me					
8. The LETHE app was hard to use					
9. I felt confident using the LETHE app					
10. I will need to learn a lot before using the LETHE app.					

Thank you for filling in this questionnaire!
Check the LETHE dashboard to see if there are any other questionnaires open.

German:
Erfahrungen mit der LETHE App

In dem folgenden Fragebogen werden Ihnen Fragen zu Ihren Erfahrungen mit der LETHE-App gestellt. Bitte lesen Sie die Fragen und die dazugehörigen Anweisungen sorgfältig durch, bevor Sie sie beantworten. Der Fragebogen besteht aus 10 Fragen, deren Beantwortung etwa 2 Minuten in Anspruch nehmen sollte.

	Stimme überhaupt nicht zu - 1	2	3	4	Stimme voll und ganz zu - 5
1. Ich würde die LETHE App häufig benutzen					
2. Die LETHE App ist für mich zu komplex					
3. Die LETHE App war einfach zu bedienen					
4. Ich brauche wirklich Hilfe von jemandem, um die LETHE App zu benutzen					
5. Die verschiedenen Aspekte der LETHE App waren gut integriert					
6. Die LETHE App war für mich verwirrend					
7. Ich konnte die Benutzung der LETHE schnell erlernen					
8. Die LETHE App war schwer zu bedienen					
9. Ich fühlte mich sicher im Umgang mit der LETHE App					
10. Ich werde noch viel lernen müssen, bevor ich die LETHE App benutze.					

Vielen Dank, dass Sie diesen Fragebogen ausgefüllt haben!
Schauen Sie in der Hauptseite nach, ob noch andere Fragebögen offen sind.

Finnish:
Kokemuksia LETHE-mobiilisovelluksesta

Tällä lomakkeella kysytään kokemuksistanne liittyen LETHE-mobiilisovellukseen. Lukekaa kysymys ja mahdolliset ohjeet huolellisesti ennen vastaamista. Lomakkeessa on yhteensä 10 kysymystä, ja sen täyttämiseen kuluu aikaa noin 2 minuuttia.

	Ehdottomasti eri mieltä - 1	2	3	4	Ehdottomasti samaa mieltä - 5
1. Luulen, että haluaisin käyttää tätä sovellusta usein					
2. Sovellus on mielestäni tarpeettoman monimutkainen					
3. Sovellus on mielestäni helppokäyttöinen					
4. Luulen, että tarvitsisin teknisen asiantuntijan tukea, jotta osaisin käyttää sovellusta					
5. Sovelluksen eri osiot/toiminnot on yhdistetty hyvin toisiinsa					
6. Sovellus on mielestäni epäjohdonmukainen					
7. Kuvittelisin, että useimmat oppisivat käyttämään sovellusta erittäin nopeasti					
8. Sovelluksen käyttö on mielestäni hankalaa/vaivalloista					
9. Tunnen oloni varmaksi sovellusta käyttäessä					
10. Minun piti opetella paljon asioita ennen kuin sovelluksen käyttö alkoi sujua					

Kiitos lomakkeen täyttämisestä!
Tarkistakaa pääsivulta, onko Teillä muita avoimia kysymyslomakkeita.

Swedish:
Användbarheten av LETHE mobilappen

Följande frågor handlar om dina erfarenheter av användandet av LETHE appen. Det finns 10 frågor och det tar ungefär 2 minuter att svara på alla frågor.

	Instämmer inte alls - 1	Instämmer inte helt	Varken instämmer eller instämmer inte	Instämmer delvis	Instämmer helt - 5
1. Jag använder gärna LETHE mobilappen					
2. Jag drar mig för att använda LETHE mobilappen, den är onödigt komplicerad					
3. Jag tycker att LETHE mobilappen är lätt att använda					
4. Jag behöver ofta hjälp av en tekniskt kunnig person för att kunna använda LETHE mobilappen					
5. Jag tycker att funktionerna i LETHE mobilappen är väl organiserade och tydliga					
6. Jag tycker att det finns för mycket inkonsekvens och ologiska vägar i LETHE mobilappen					
7. Jag kan tänka mig att de flesta skulle lära sig att använda LETHE mobilappen mycket snabbt					
8. Jag tror att många tycker att LETHE mobilappen är mycket besvärligt att använda					
9. Jag känner mig väldigt säker på hur jag skall använda LETHE mobilappen					
10. Jag behövde lära mig mycket innan jag kom igång med LETHE mobilappen					

Tack för att du fyllde i detta formulär!
Kontrollera i översikten om det finns fler öppna frågeformulär.

Italian:
Esperienza con l’app Lethe

Nel seguente questionario ti verranno poste alcune domande riguardanti la tua esperienza con l'app LETHE. Si prega di leggere attentamente le domande e le relative istruzioni prima di rispondere. Il questionario è composto da 10 domande, la cui risposta dovrebbe richiedere circa 2 minuti.

	Fortemente in disaccordo - 1	2	3	4	Fortemente d’accordo - 2
1. Penso che mi piacerebbe usare frequentemente l'app LETHE					
2. Ho trovato l'app LETHE inutilmente complessa					
3. Ho pensato che L'app LETHE fosse facile da usare					
4. Penso che avrei bisogno del supporto di una persona che già la conosce per poter utilizzare l'app LETHE					
5. Ho scoperto che le varie funzioni dell'app LETHE erano ben integrate					
6. Ho pensato che ci fosse troppa incoerenza nelle funzionalità dell'app LETHE					
7. Immagino che la maggior parte delle persone imparerebbe a usare l'app LETHE molto rapidamente					
8. Ho trovato L'app LETHE molto macchinosa da usare					
9. Mi sentivo molto sicuro di usare l'app LETHE					
10. Ho avuto bisogno di imparare molto prima di usare l'app LETHE.					

Grazie per aver completato il questionario!
Torna alla pagina principale per vedere se ci sono altri questionari disponibili
