# Supplementary material for: Design of a Mobile App and a Clinical Trial Management System for Cognitive Health and Dementia Risk Reduction: User-Centered Design Approach
Source: JMIR Aging. 2025 Jul 2;8:e66660. doi: 10.2196/66660 (PMC12268216; doi:10.2196/66660)
Supplement: Multimedia Appendix 2 [file aging_v8i1e66660_app2.pdf]

This is a Multimedia Appendix to a full manuscript published in the J Med Internet Res. For full copyright and citation information see http://dx.doi.org/10.2196/jmir.66660

Feedback Dashboard

This survey will just take a few minutes to complete, and your responses will remain completely confidential.
We appreciate your participation in the survey! The results of the survey will be published in a paper.
We use the results to improve the Dashboard as well.
Thank you for your participation!

* Erforderlich

Role in the Project and in the Dashboard

1

Could you please describe your role in the project? *

- ◯ Study Nurse
- ◯ Nutritionist
- ◯ Neuropsychologist
- ◯ Physiotherapist
- ◯ Sonstiges

2

What Dashboard role do you have? *

We have different roles in the Dashboard. If you are not sure which role you have, we have a short guide:

Coordinator role: has access to the whole Dashboard functionalities including the overview page with the study participants from all sites, the detailed view of a participant, the electronic case report form (eCRF, clinical data entry page in the Dashboard) and the configuration pages for the Educational Content and the videos.

Blinded role: only has access to the overview page with the study participants from his/her side and the eCRF

Unblinded role: has access to the overview page with the study participants from his/her side, the detailed view of a participant and the eCRF

- ◯ Coordinator role
- ◯ Blinded role
- ◯ Unblinded role

General Questions

3

The Dashboard has an easy onboarding process:

	Strongly agree	Agree	Neither agree or disagree	Disagree	Strongly disagree
The Dashboard has an easy onboarding process:	○	○	○	○	○

4

What specific aspects contribute to your rating above? Do you have suggestions to improve the rating?

5

How frequently do you access the Dashboard?

- ◯ Daily
- ◯ Once a week
- ◯ More than once a week
- ◯ More than once a month
- ◯ Once a month

6

The Dashboard is easy to use:

	Strongly agree	Agree	Neither agree or disagree	Disagree	Strongly disagree
The Dashboard is easy to use:	◯	◯	◯	◯	◯

7

What specific aspects contribute to your rating above? Do you have suggestions to improve the rating?

8

It is easy to navigate through the Dashboard:

	Strongly agree	Agree	Neither agree or disagree	Disagree	Strongly disagree
It is easy to navigate through the Dashboard:	○	○	○	○	○

9

What specific aspects contribute to your rating above? Do you have suggestions to improve the rating?

10

I like the overall design of the Dashboard:

	Strongly agree	Agree	Neither agree or disagree	Disagree	Strongly disagree
I like the overall design of the Dashboard:	○	○	○	○	○

11

What specific aspects contribute to your rating above? Do you have suggestions to improve the rating?

Overview Page

The following question refers to the Overview Page of the Dashboard as shown in the picture.

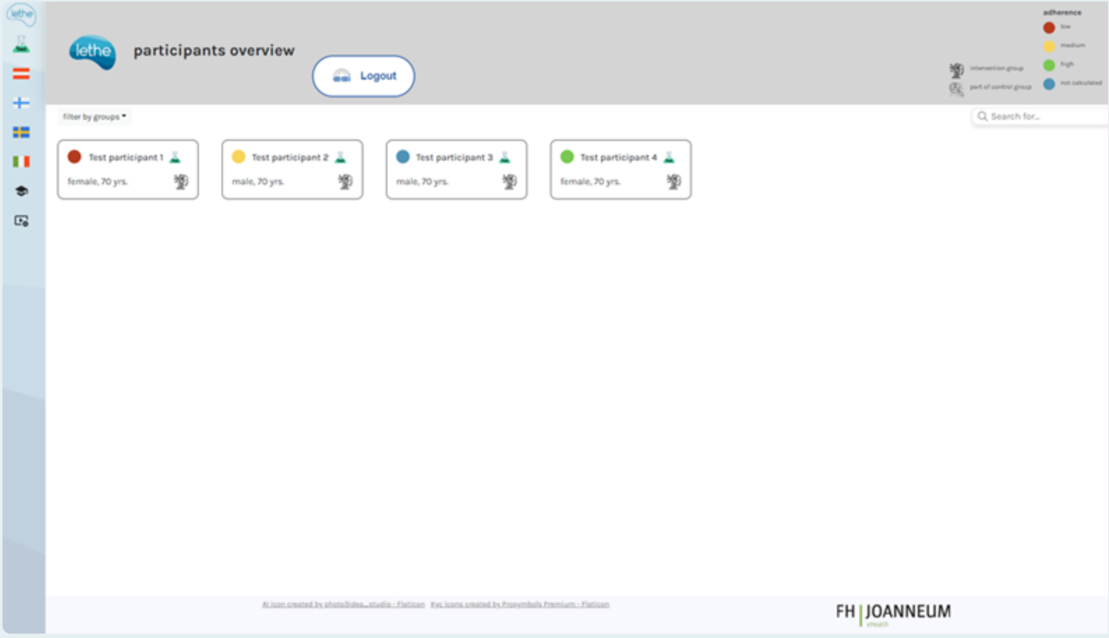

12

The overview page displays all relevant information about each participant in an easily understandable way:

	Strongly agree	Agree	Neither agree or disagree	Disagree	Strongly disagree
The overview page displays all relevant information about each participant in an easily understandable way:	○	○	○	○	○

13

What specific aspects contribute to your rating above? Do you have suggestions to improve the rating?

Configuration Pages

The following question referss to the Configuration Pages of the Dashboard as shown in the picture. If you do not have access to the Configuration Pages, you can skip this section.

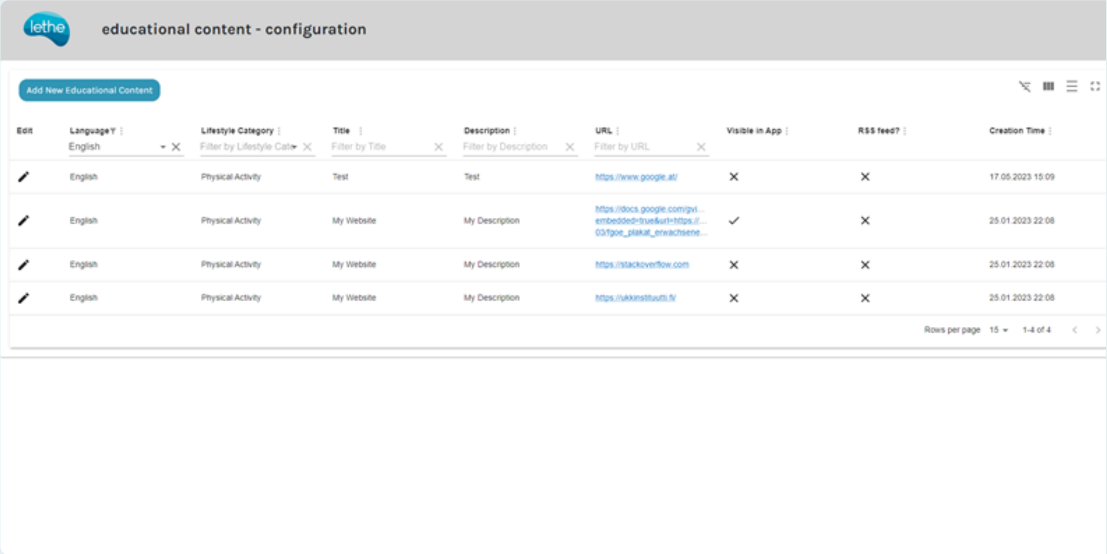

14

I think the configuration of the Educational Content for the LETHE App is straightforward:

	Strongly agree	Agree	Neither agree or disagree	Disagree	Strongly disagree
I think the configuration of the Educational Content for the LETHE App is straightforward:	◯	◯	◯	◯	◯

15

What specific aspects contribute to your rating above? Do you have suggestions to improve the rating?

16

I think the configuration of the Videos for the LETHE App is straightforward:

	Strongly agree	Agree	Neither agree or disagree	Disagree	Strongly disagree
I think the configuration of the Videos for the LETHE App is straightforward:	○	○	○	○	○

17

What specific aspects contribute to your rating above? Do you have suggestions to improve the rating?

Detail Page of a Participant

The following questions refer to the features of the Detail Page of the Dashboard as shown in the picture. The questions include the overall experience (insertions, visualization, filtering, ...). If you do not have access to the Detail Page, you can skip this section.

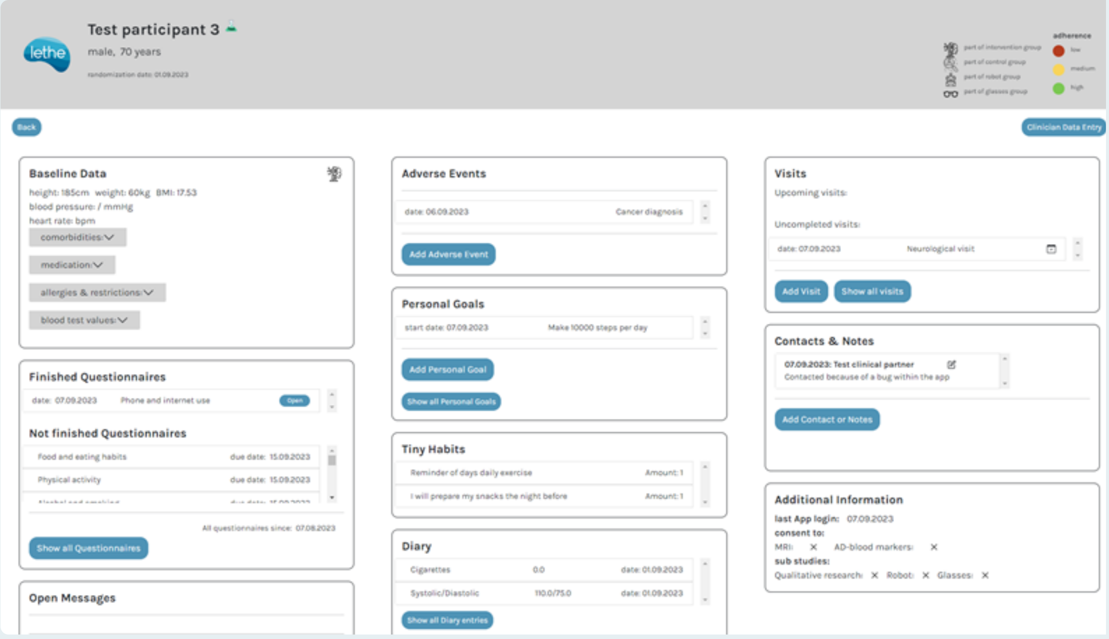

18

I think the Questionnaires and their Responses Section on the Detail Page is easy to understand:

	Strongly agree	Agree	Neither agree or disagree	Disagree	Strongly disagree
I think the Questionnaires and their Responses Section on the Detail Page is easy to understand:	○	○	○	○	○

19

What specific aspects contribute to your rating above? Do you have suggestions to improve the rating?

20

I think the Notification Section on the Detail Page is easy to understand:

	Strongly agree	Agree	Neither agree or disagree	Disagree	Strongly disagree
I think the Notification Section on the Detail Page is easy to understand:	◯	◯	◯	◯	◯

21

What specific aspects contribute to your rating above? Do you have suggestions to improve the rating?

22

I think the Adverse Event Section on the Detail Page is easy to understand/use:

	Strongly agree	Agree	Neither agree or disagree	Disagree	Strongly disagree
I think the Adverse Event Section on the Detail Page is easy to understand/use:	○	○	○	○	○

23

What specific aspects contribute to your rating above? Do you have suggestions to improve the rating?

24

I think the Personal Goal Section on the Detail Page is easy to understand/use:

	Strongly agree	Agree	Neither agree or disagree	Disagree	Strongly disagree
I think the Personal Goal Section on the Detail Page is easy to understand/use:	○	○	○	○	○

25

What specific aspects contribute to your rating above? Do you have suggestions to improve the rating?

26

I think the Diary Entries Section on the Detail Page is easy to understand:

	Strongly agree	Agree	Neither agree or disagree	Disagree	Strongly disagree
I think the Diary Entries Section on the Detail Page is easy to understand:	○	○	○	○	○

27

What specific aspects contribute to your rating above? Do you have suggestions to improve the rating?

28

I think the Visits Section on the Detail Page is easy to understand/use:

	Strongly agree	Agree	Neither agree or disagree	Disagree	Strongly disagree
I think the Visits Section on the Detail Page is easy to understand/use:	○	○	○	○	○

29

What specific aspects contribute to your rating above? Do you have suggestions to improve the rating?

30

I think the Contacts/Notes Section on the Detail Page is easy to understand/use:

	Strongly agree	Agree	Neither agree or disagree	Disagree	Strongly disagree
I think the Contacts/Notes Section on the Detail Page is easy to understand/use:	○	○	○	○	○

31

What specific aspects contribute to your rating above? Do you have suggestions to improve the rating?

Electronic Case Report Form (eCRF) for Visit Data

The following question refer to the Clinical Data Entry Page of the Dashboard.

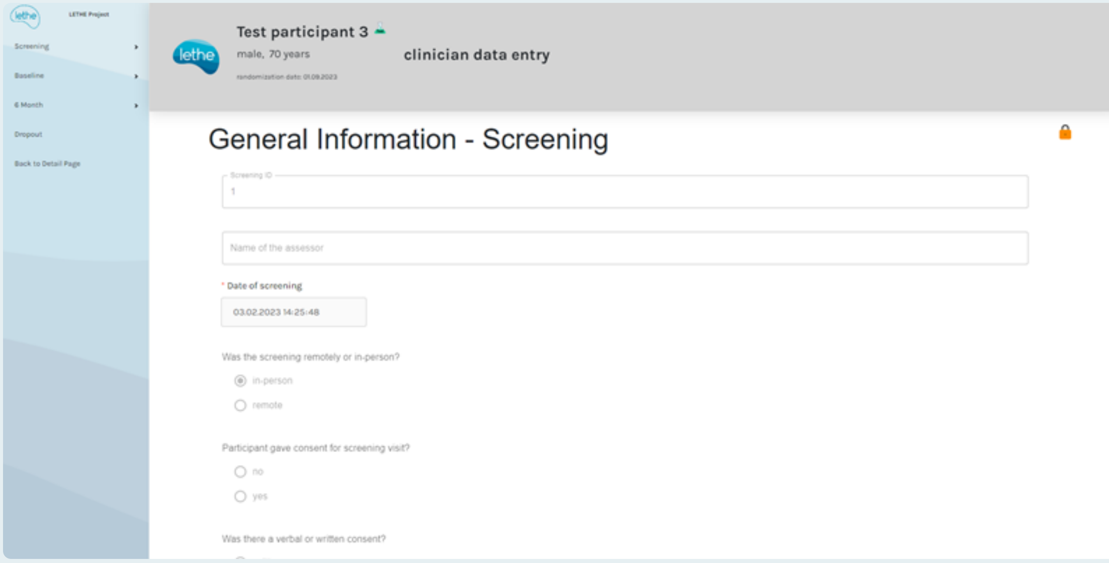

32

I think the eCRF is easy to understand/use:

	Strongly agree	Agree	Neither agree or disagree	Disagree	Strongly disagree
I think the eCRF is easy to understand/ use:	○	○	○	○	○

33

What specific aspects contribute to your rating above? Do you have suggestions to improve the rating?

New Features/Issues

34

Are there any specific features you would like to see added to the Dashboard?

35

Have you encountered any issues such as bugs or crashes while using the Dashboard? If so, could you please specify them.

36

I was satisfied with the support when encountering problems:

	Strongly agree	Agree	Neither agree or disagree	Disagree	Strongly disagree
I was satisfied with the support when encountering problems:	○	○	○	○	○

37

What specific aspects contribute to your rating above? Do you have suggestions to improve the rating?

38

The Dashboard is stable and I am not worried about making any false entries:

	Strongly agree	Agree	Neither agree or disagree	Disagree	Strongly disagree
The Dashboard is stable and I am not worried about making any false entries:	◯	◯	◯	◯	◯

39

What specific aspects contribute to your rating above? Do you have suggestions to improve the rating?

40

Which feature you consider most useful and beyond state of the art? Please rank the items below by their importance (first item = most useful and beyond state of the art; last item = least useful and not beyond state of the art).

- Real time planning of appointments within the Dashboard for the App
- Integration of personalized intervention tasks (e.g. Personal Goals)
- eCRF with automatic calculations
- Configuration of the content of the App via the Dashboard
- Immediate results of onboarding and questionnaires in the Dashboard
- Direct notifications to study participants via the Dashboard
- Overview page of different study participants in different countries including their Digital Intervention Pathways

Additional Comments

41

Please feel free to share any additional comments or feedback regarding your experience with the Dashboard.

Dieser Inhalt wurde von Microsoft weder erstellt noch gebilligt. Die von Ihnen übermittelten Daten werden an den Formulareigentümer gesendet.

Microsoft Forms
