# Supplementary material for: Design of a Mobile App and a Clinical Trial Management System for Cognitive Health and Dementia Risk Reduction: User-Centered Design Approach
Source: JMIR Aging. 2025 Jul 2;8:e66660. doi: 10.2196/66660 (PMC12268216; doi:10.2196/66660)
Supplement: Multimedia Appendix 3 [file aging_v8i1e66660_app3.pptx]

## Slide 1
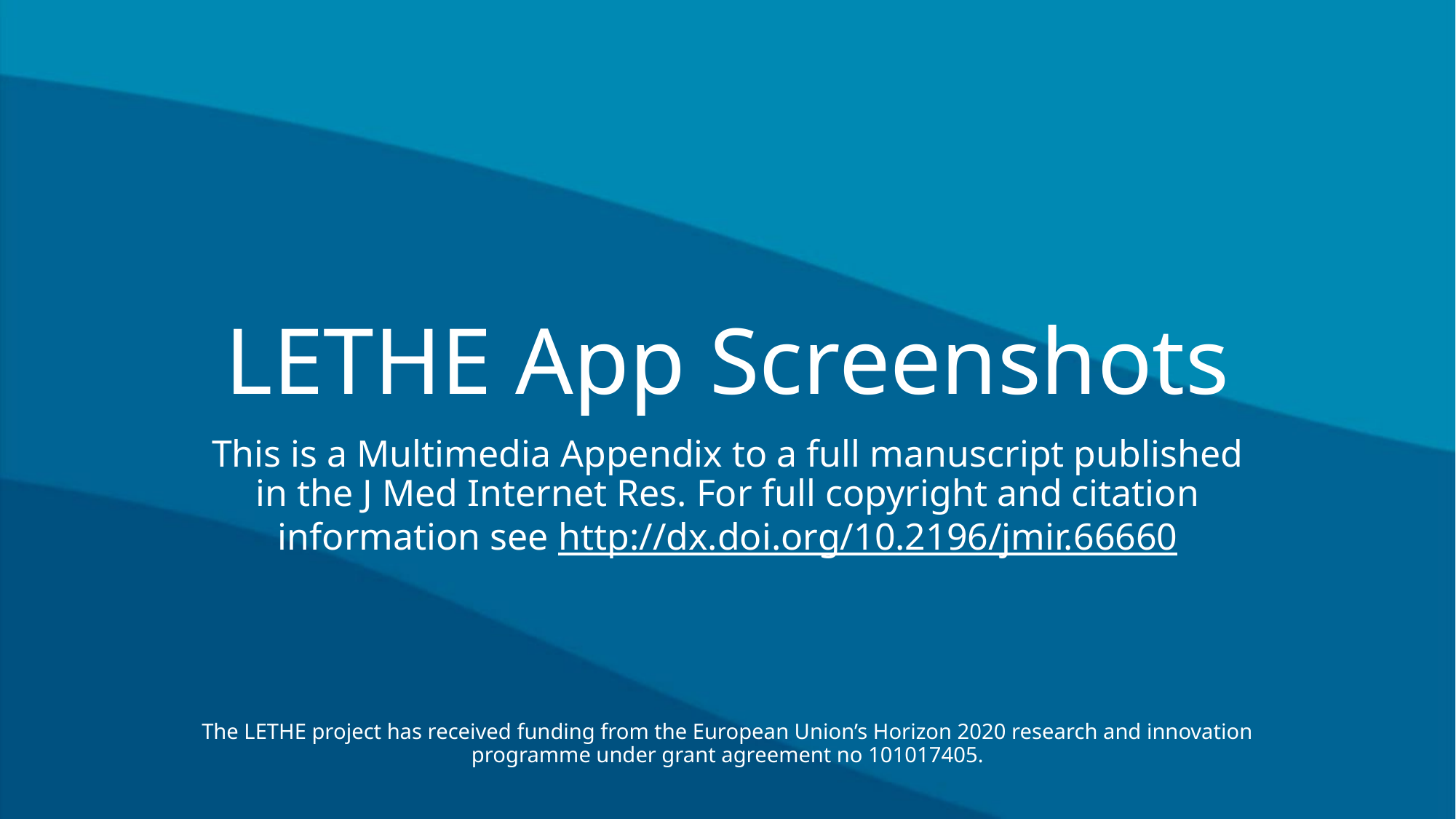

# LETHE App Screenshots
This is a Multimedia Appendix to a full manuscript published in the J Med Internet Res. For full copyright and citation information see http://dx.doi.org/10.2196/jmir.66660
The LETHE project has received funding from the European Union’s Horizon 2020 research and innovation programme under grant agreement no 101017405.

## Slide 2
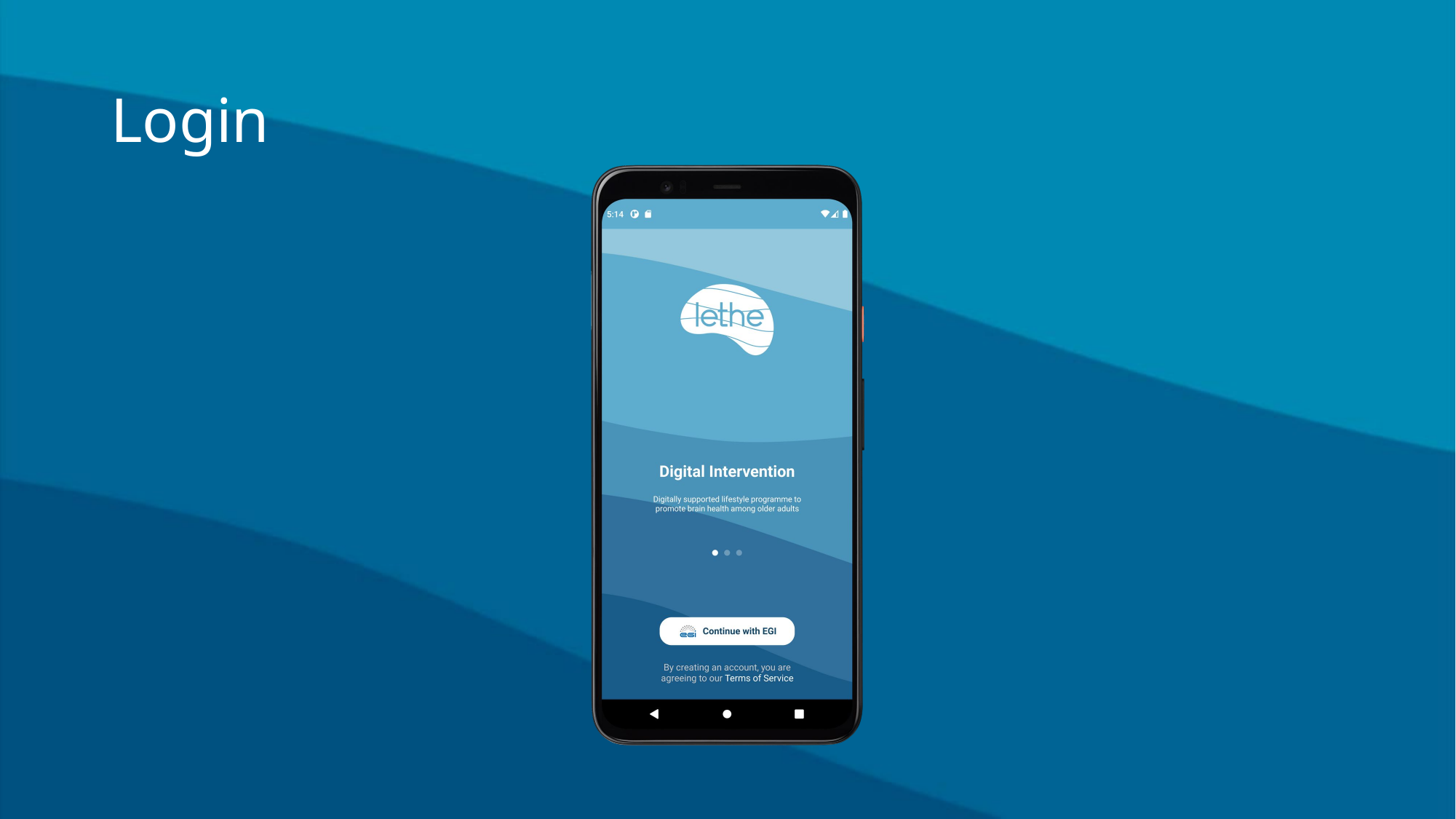

# Login

## Slide 3
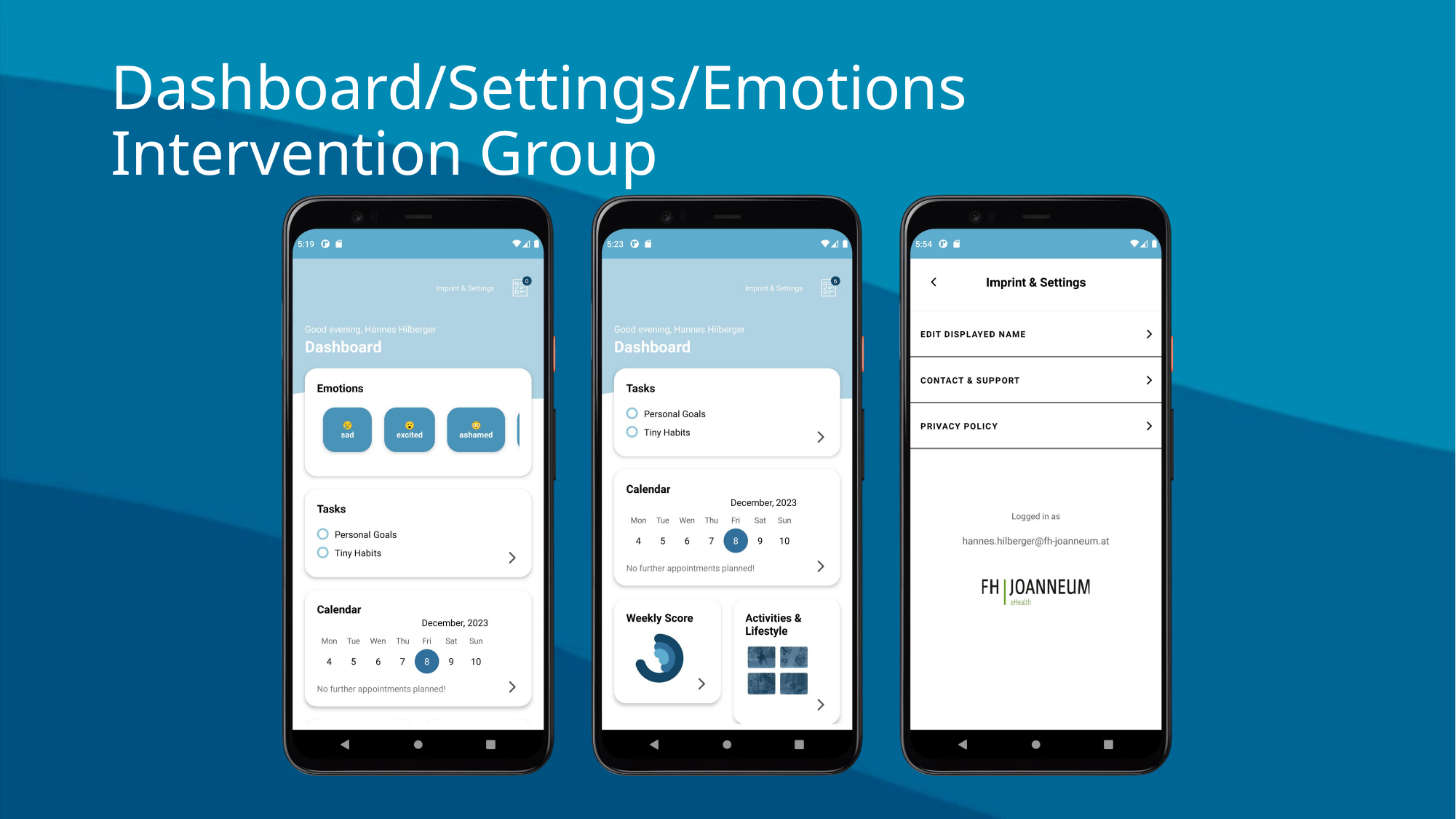

# Dashboard/Settings/Emotions Intervention Group

## Slide 4
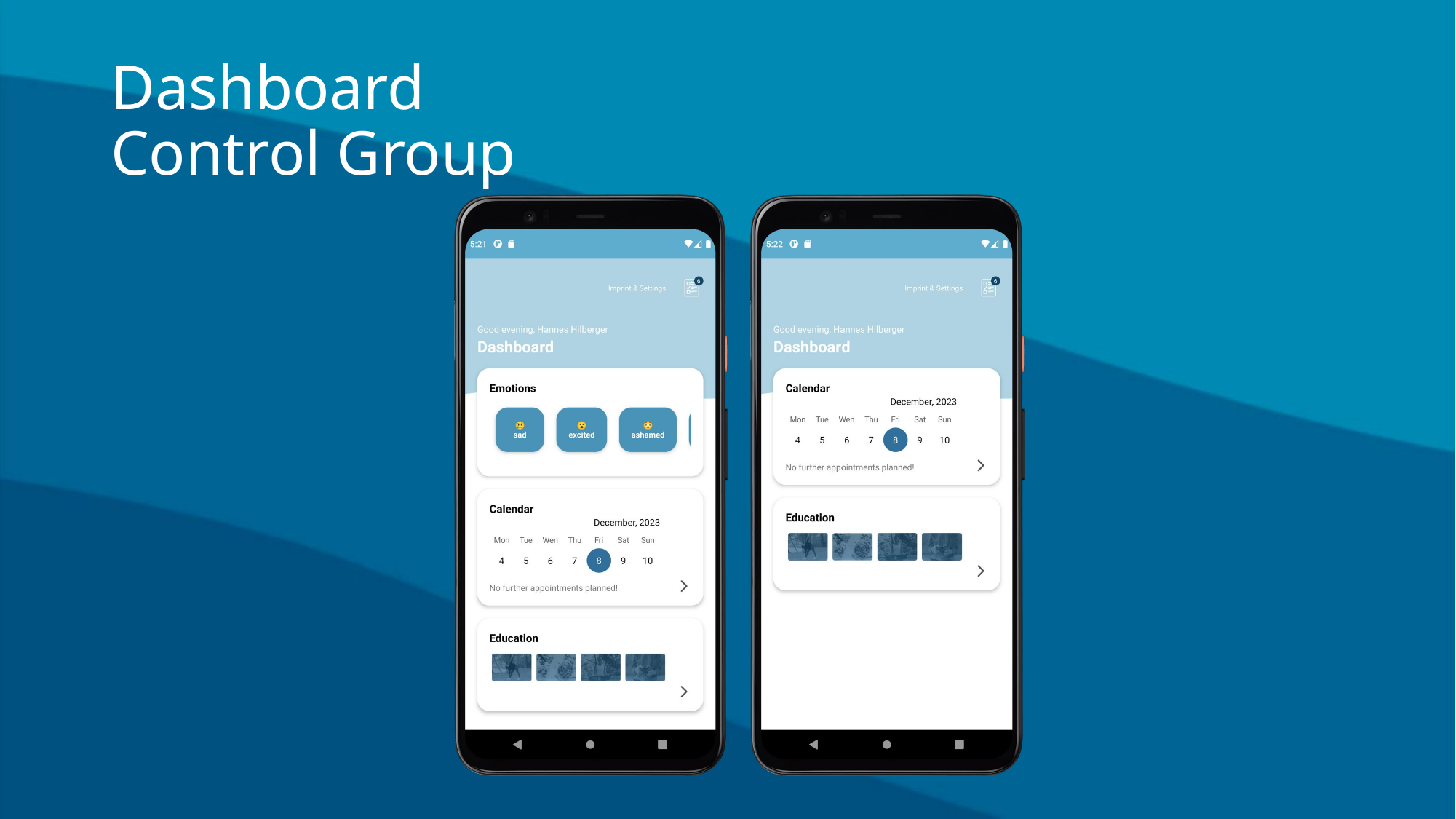

# Dashboard Control Group

## Slide 5
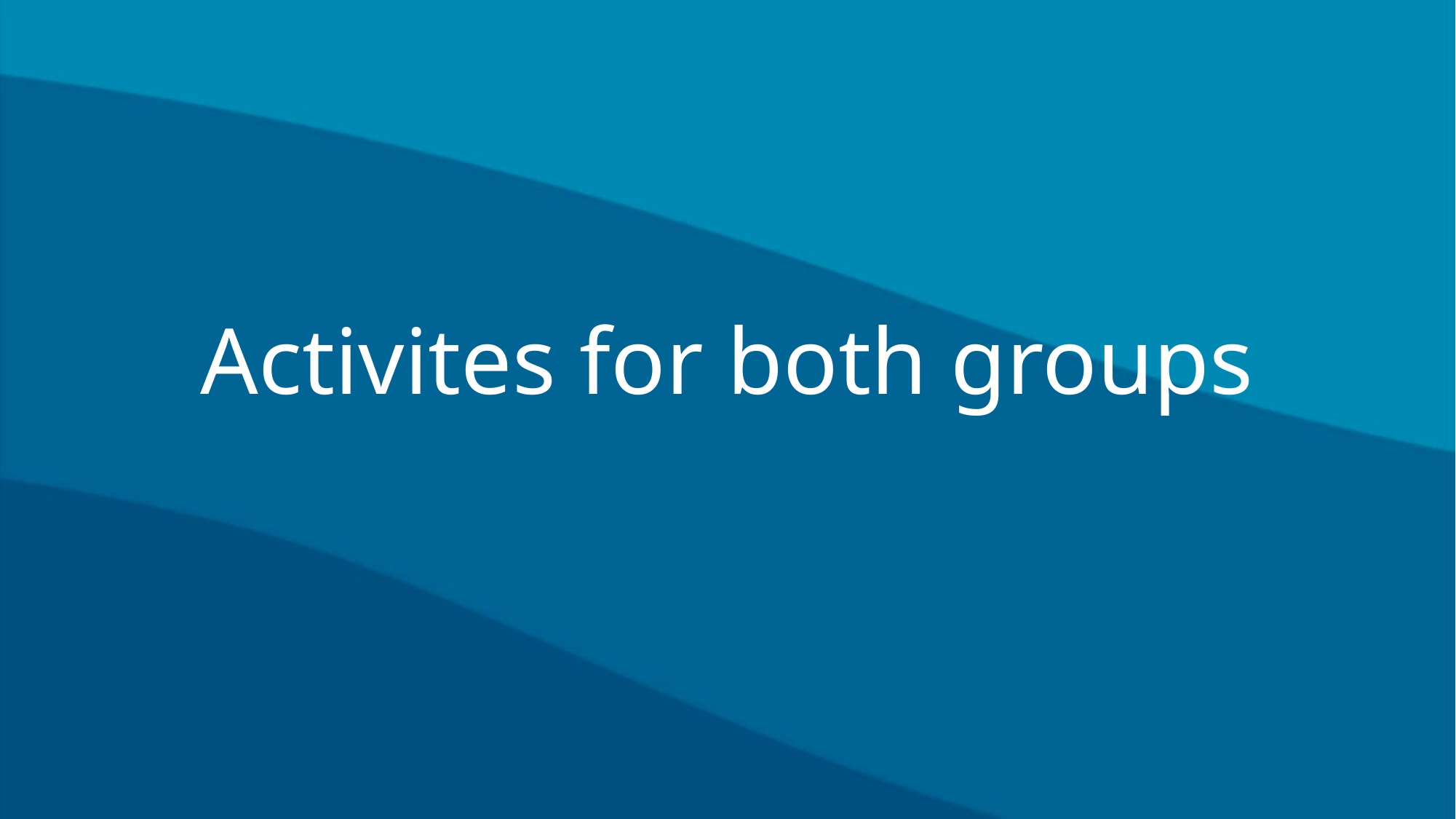

# Activites for both groups

## Slide 6
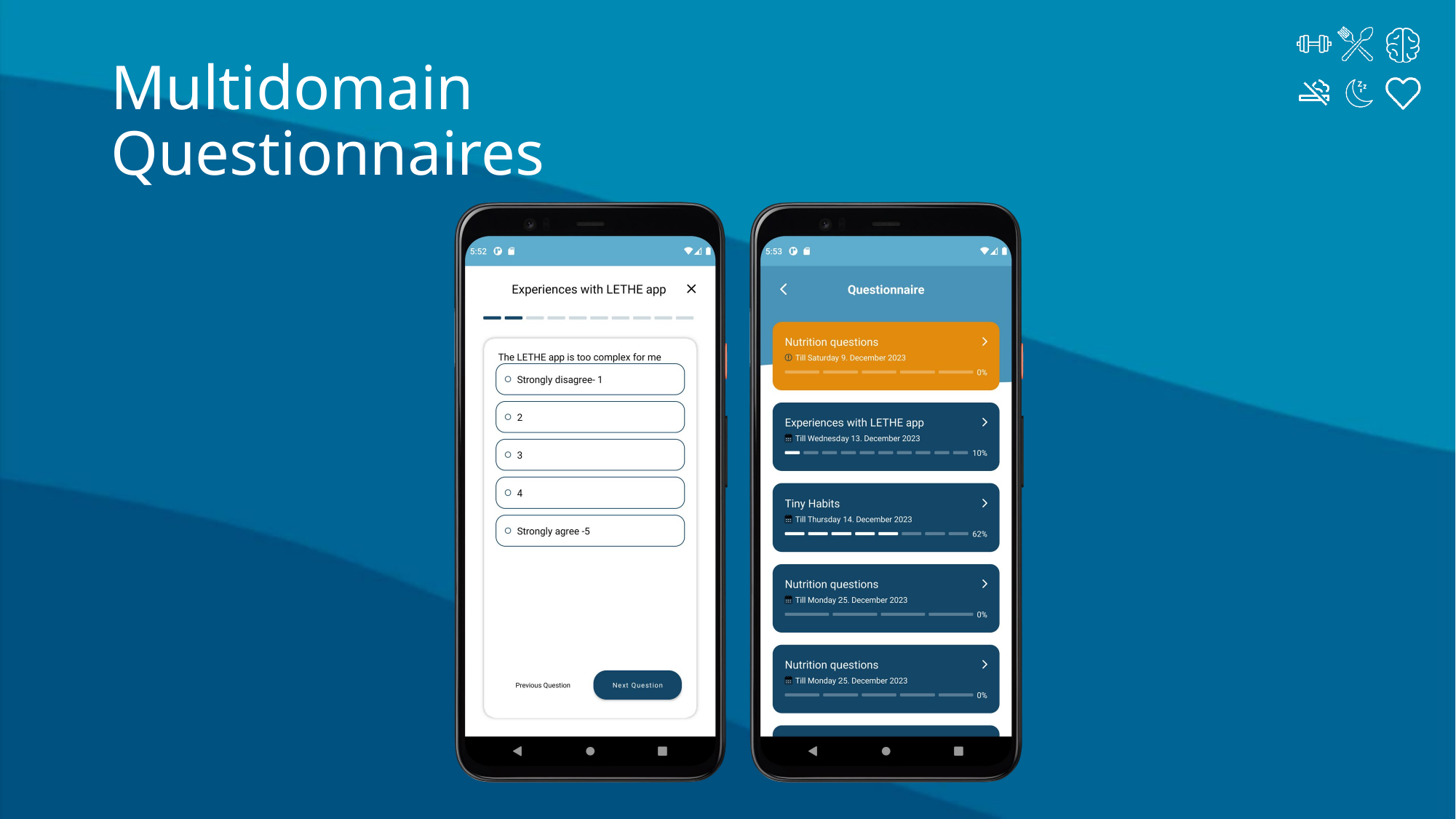

# Multidomain Questionnaires

## Slide 7
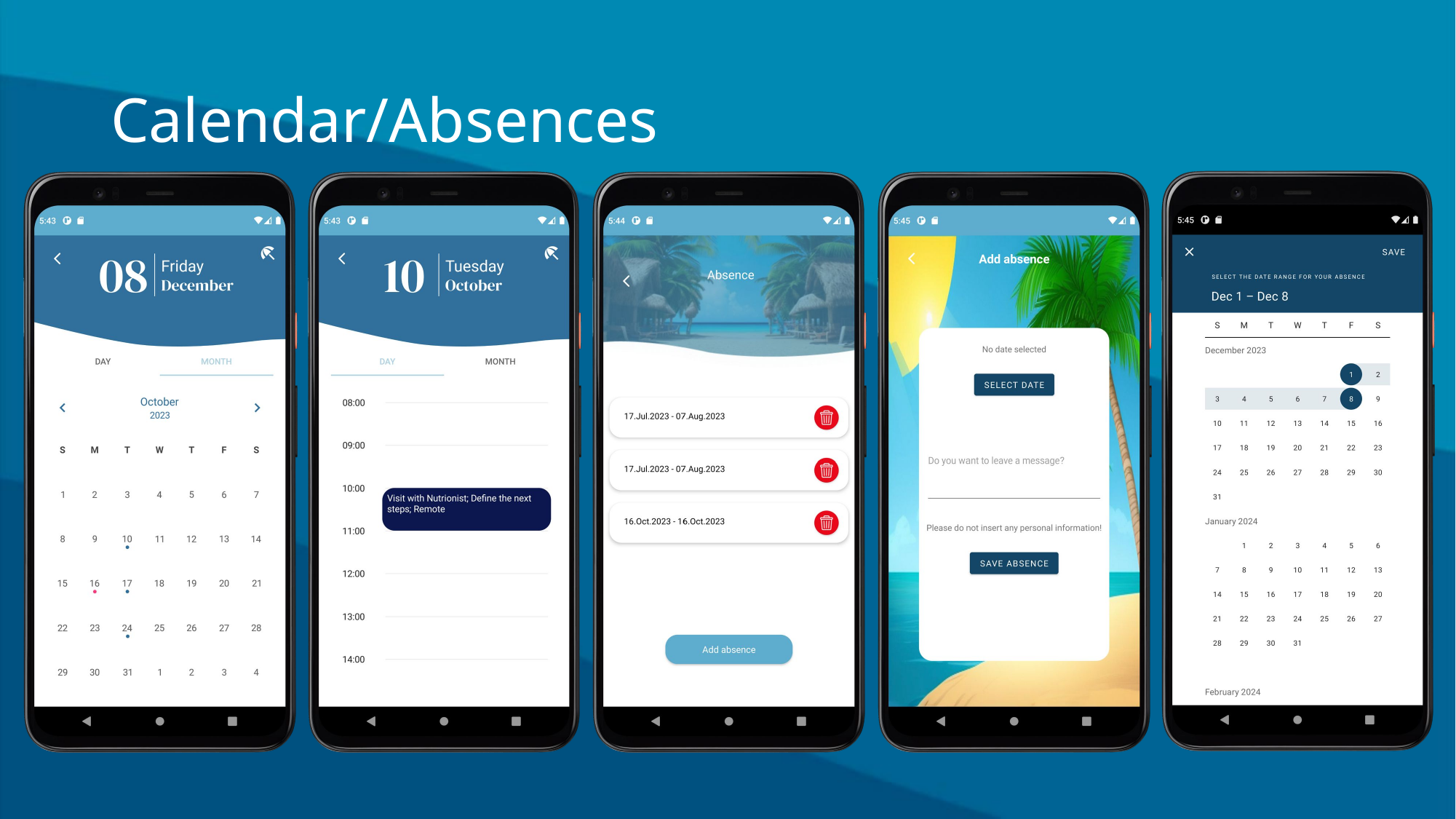

# Calendar/Absences

## Slide 8
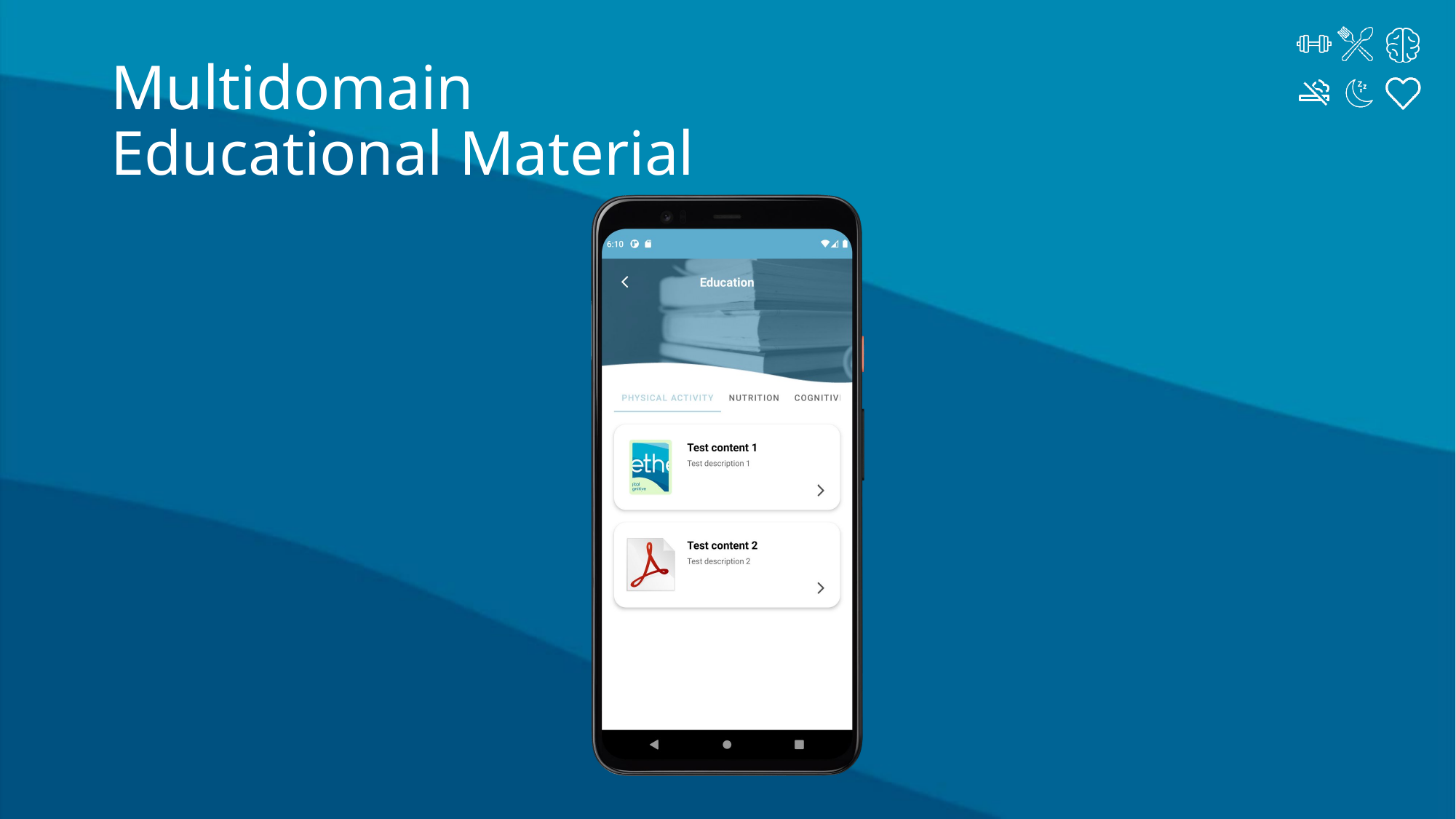

# Multidomain Educational Material

## Slide 9
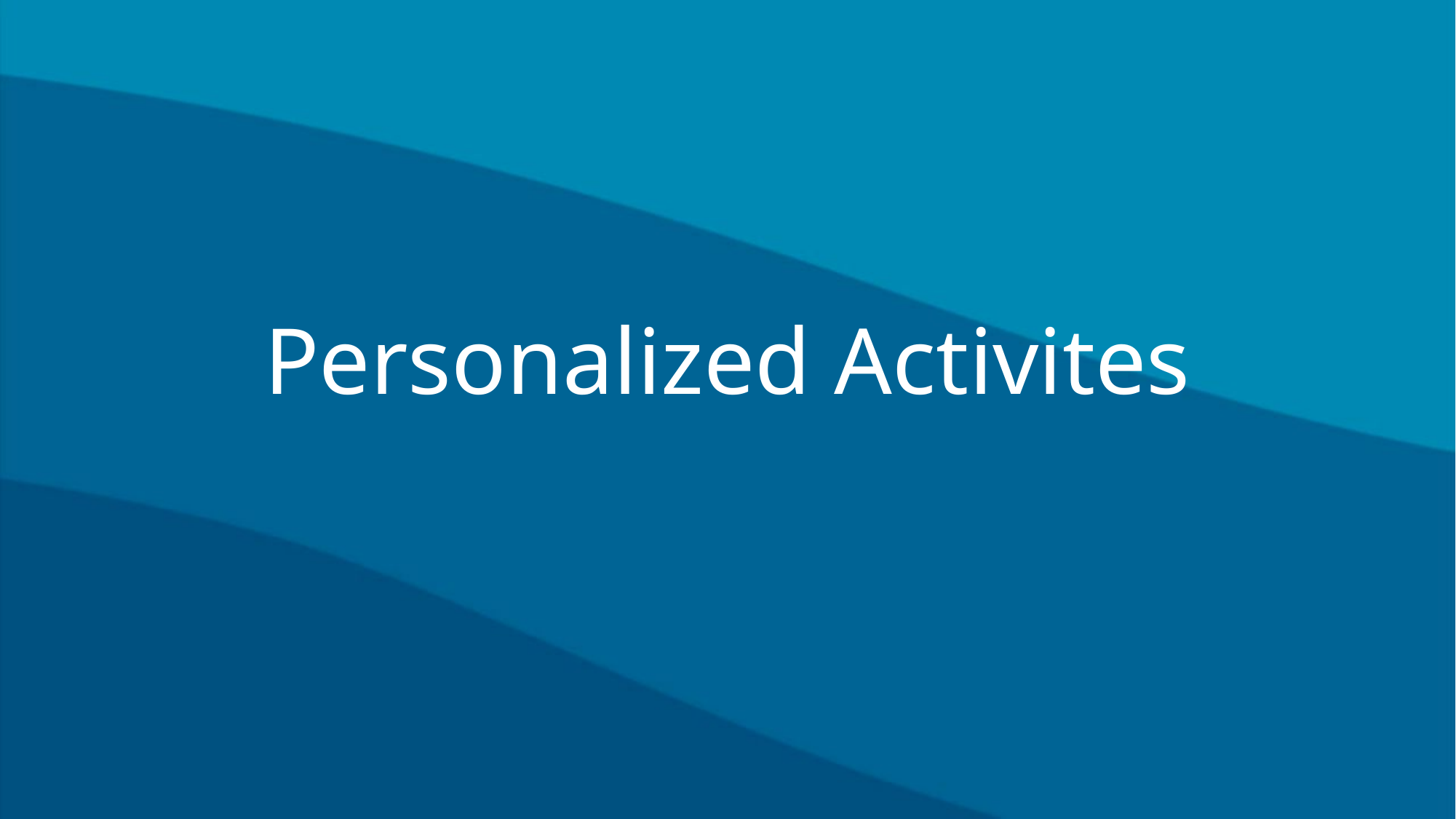

# Personalized Activites

## Slide 10
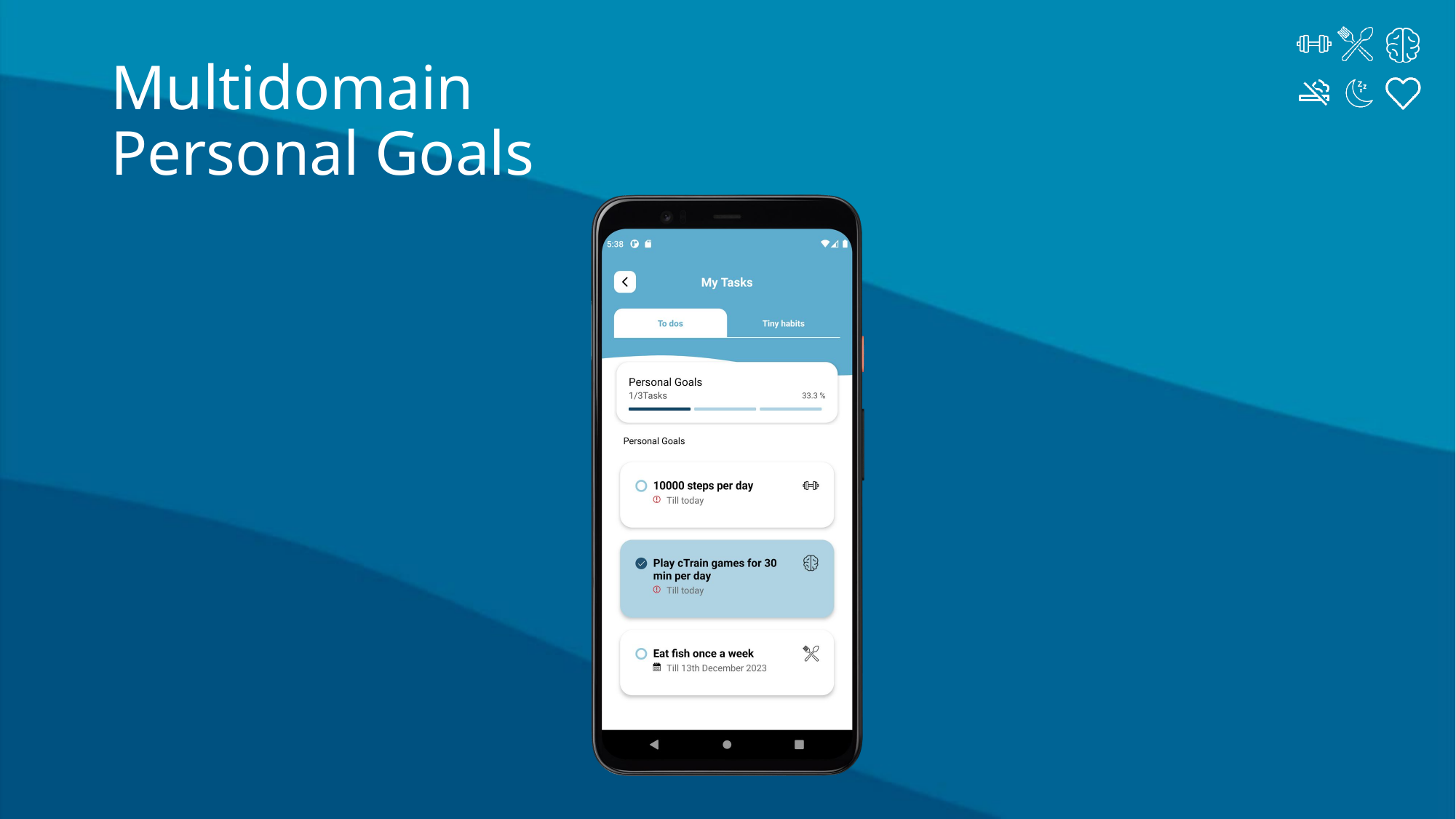

# Multidomain Personal Goals

## Slide 11
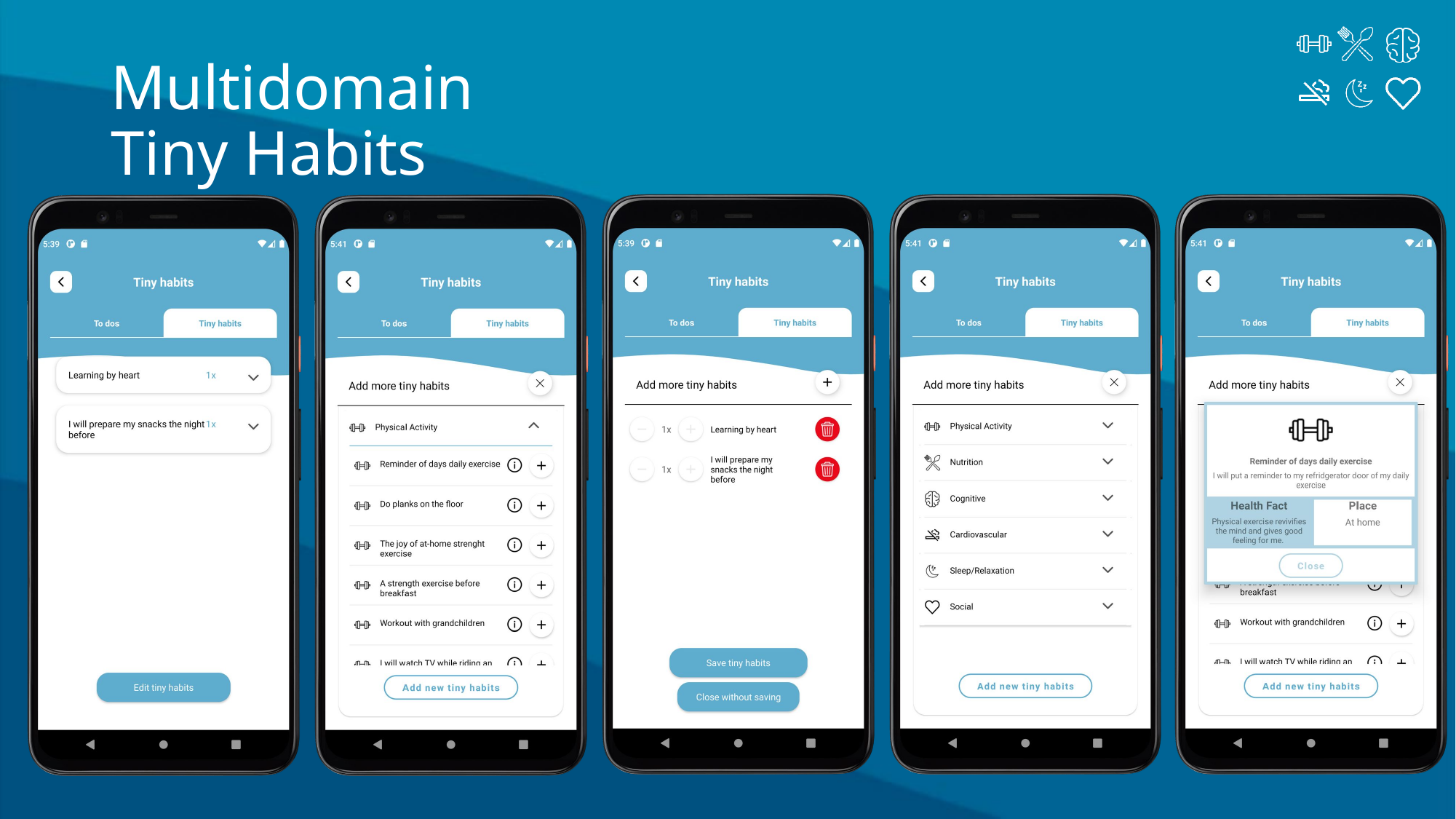

# Multidomain Tiny Habits

## Slide 12
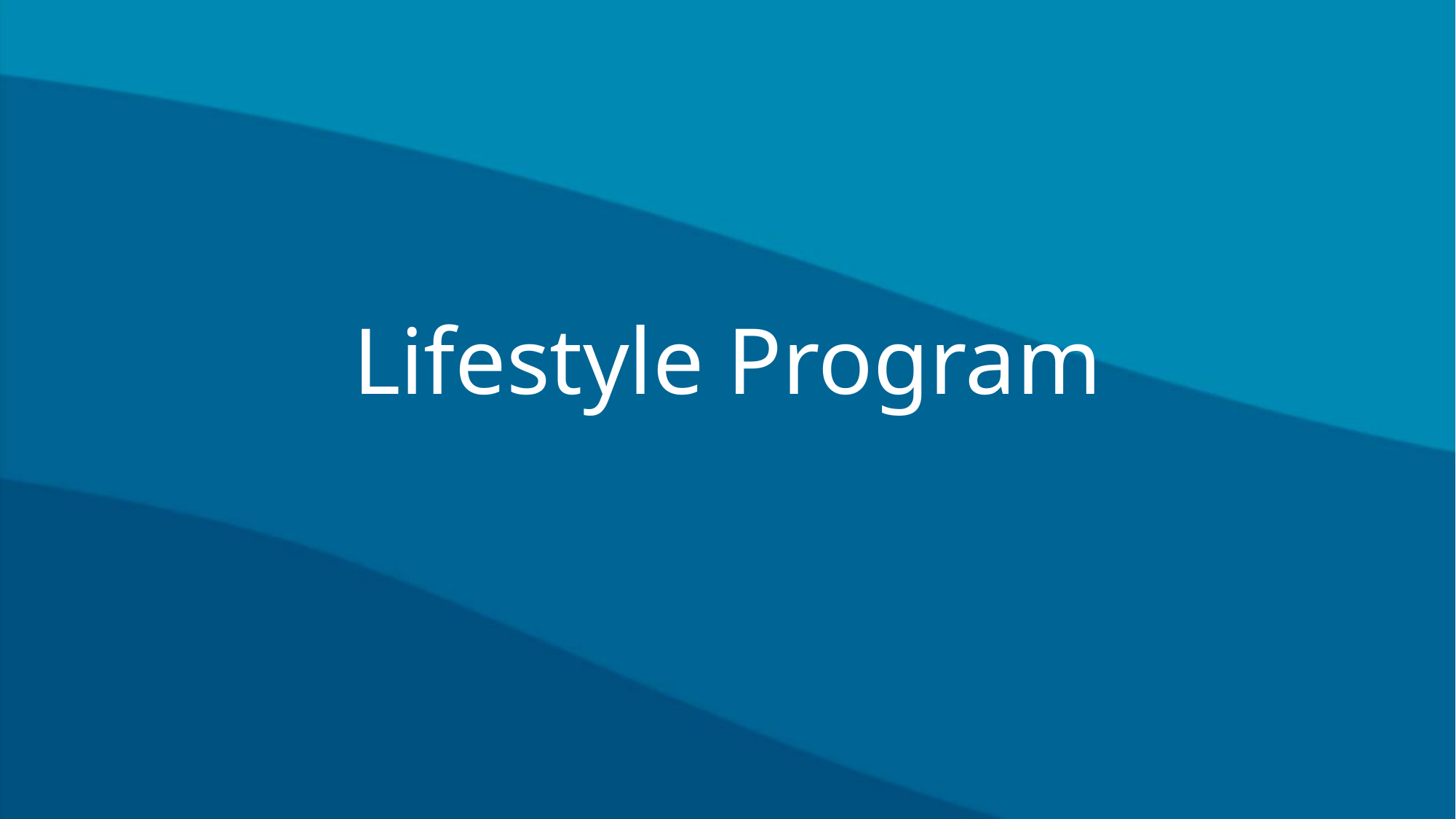

# Lifestyle Program

## Slide 13
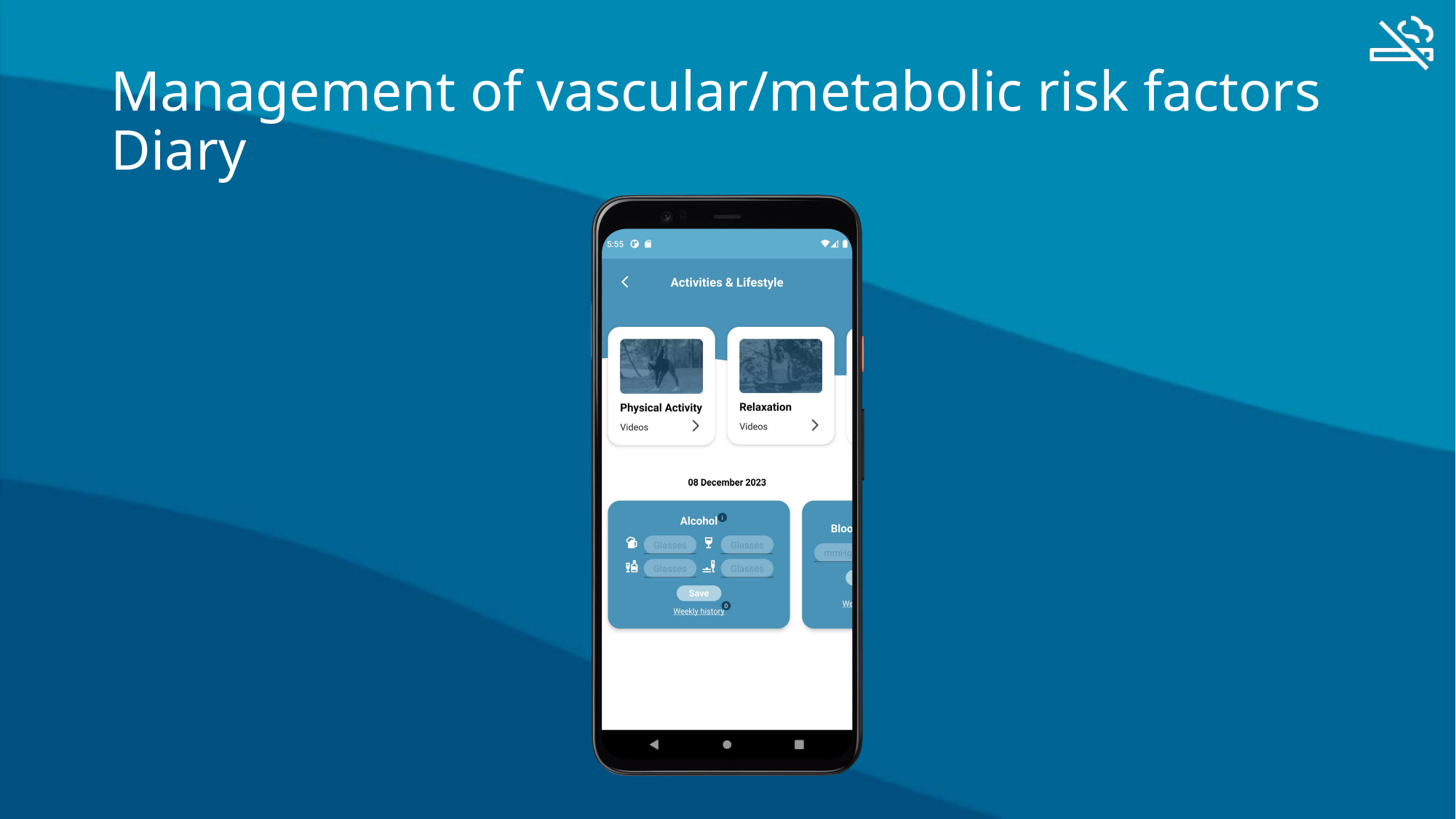

# Management of vascular/metabolic risk factors Diary

## Slide 14
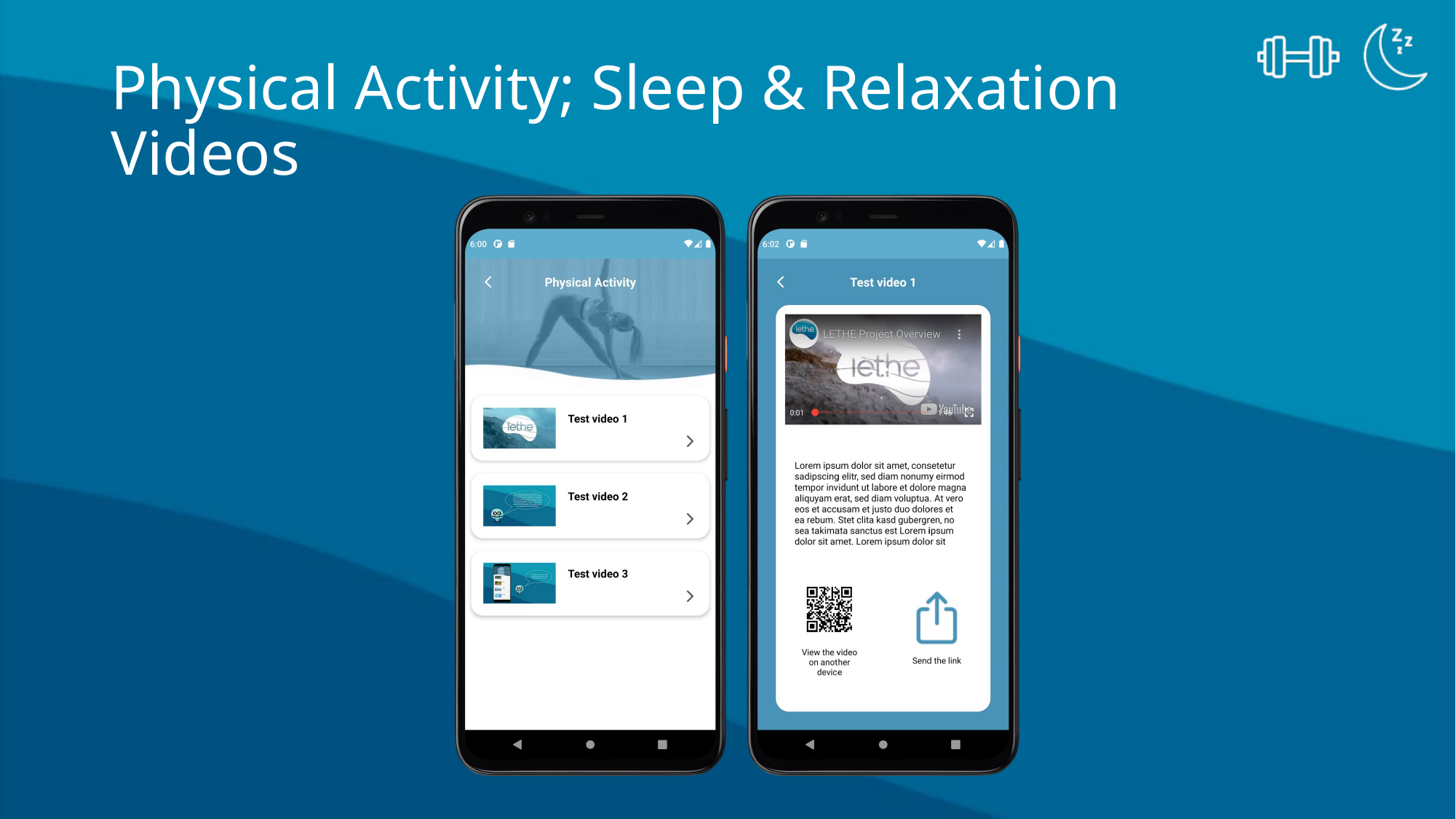

# Physical Activity; Sleep & Relaxation Videos

## Slide 15
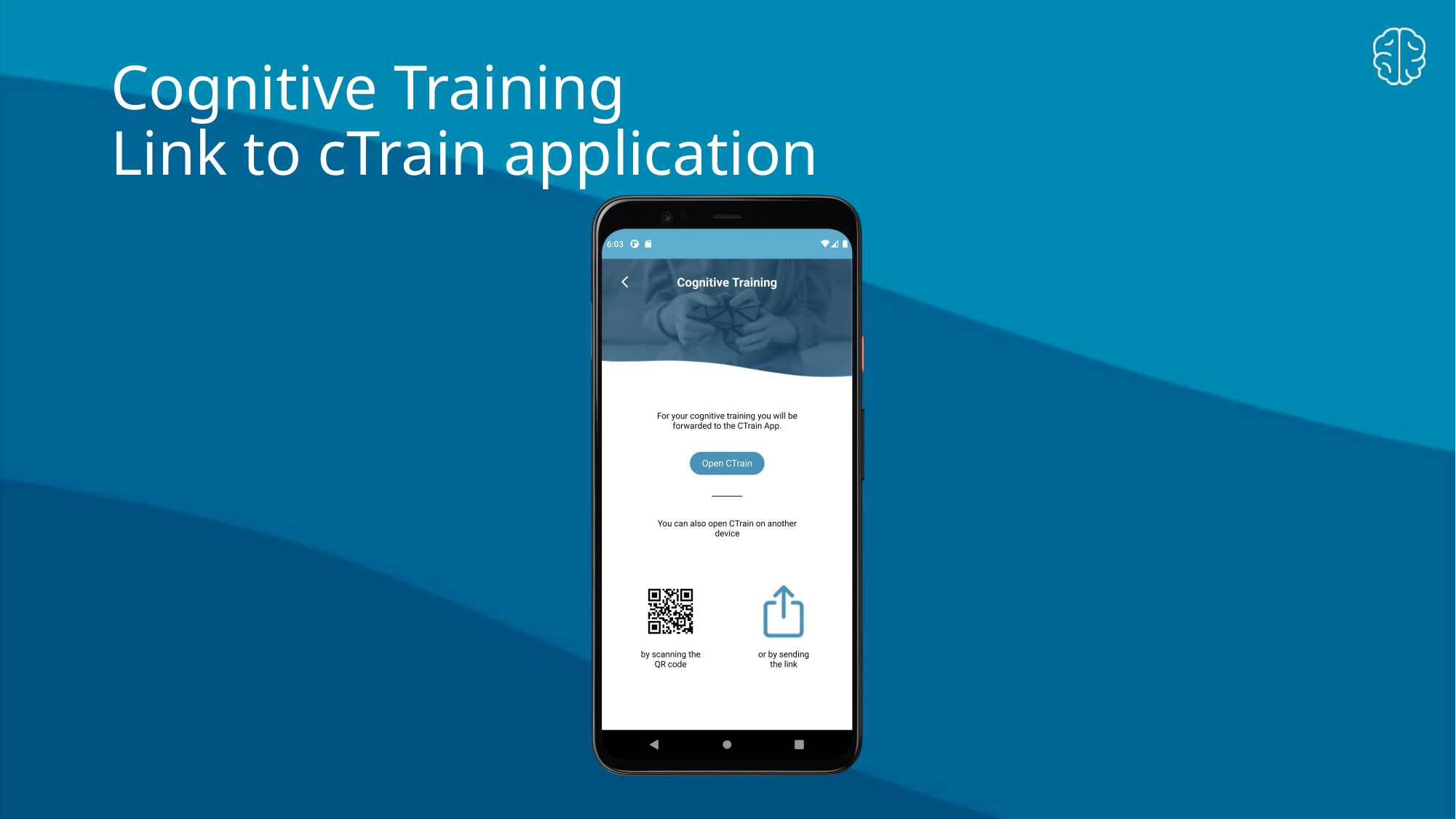

# Cognitive Training Link to cTrain application

## Slide 16
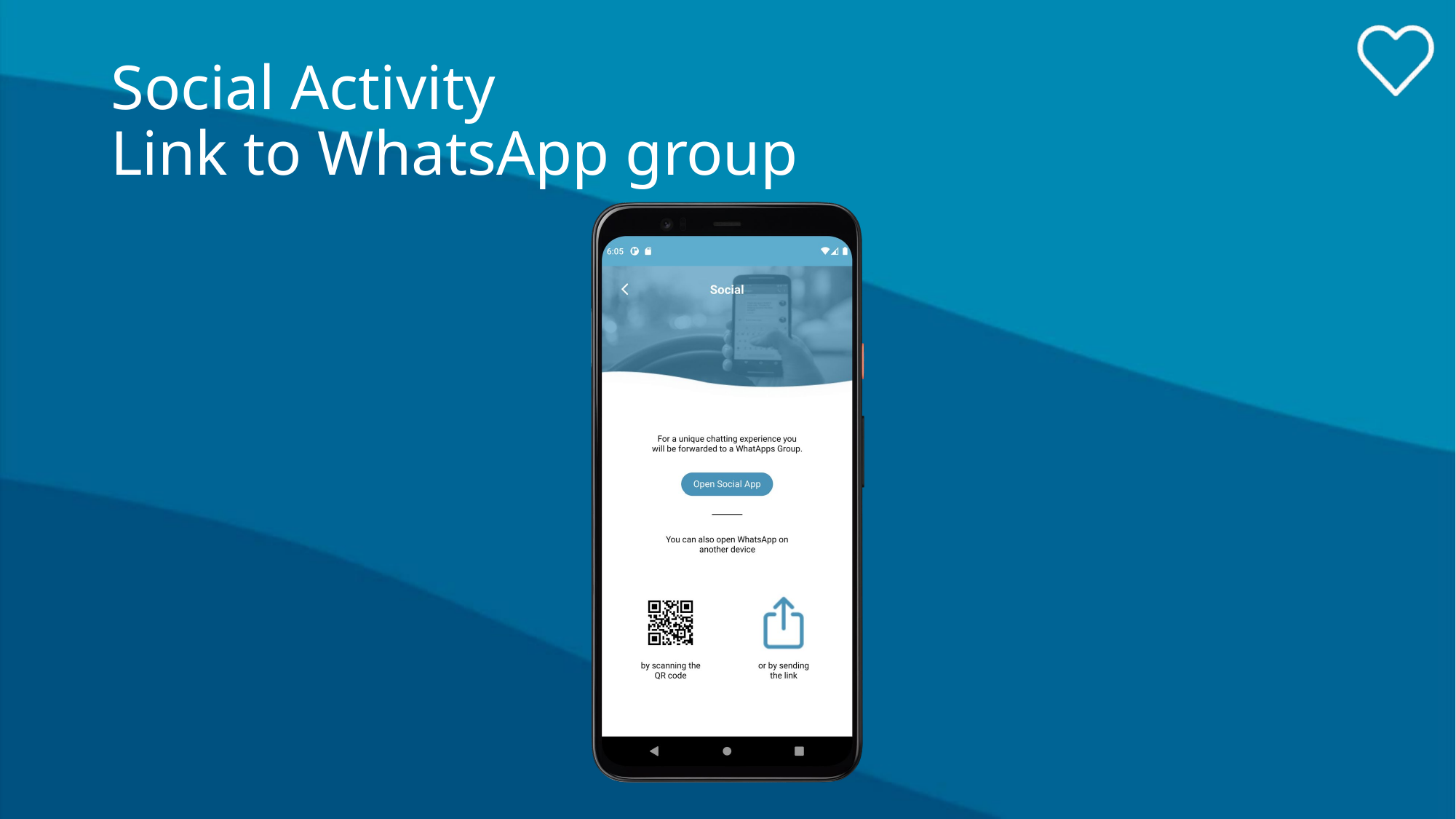

# Social Activity Link to WhatsApp group

## Slide 17
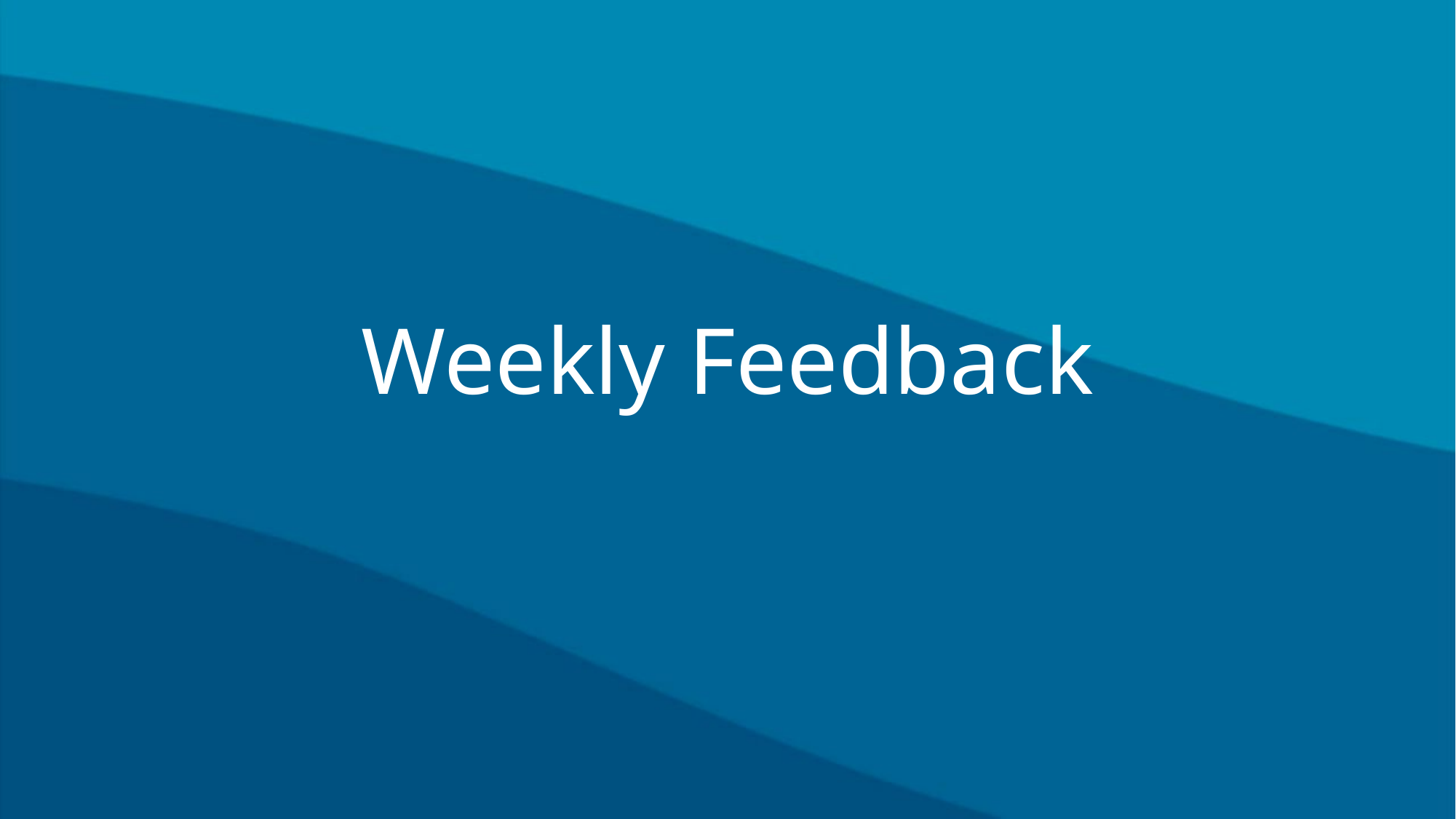

# Weekly Feedback

## Slide 18
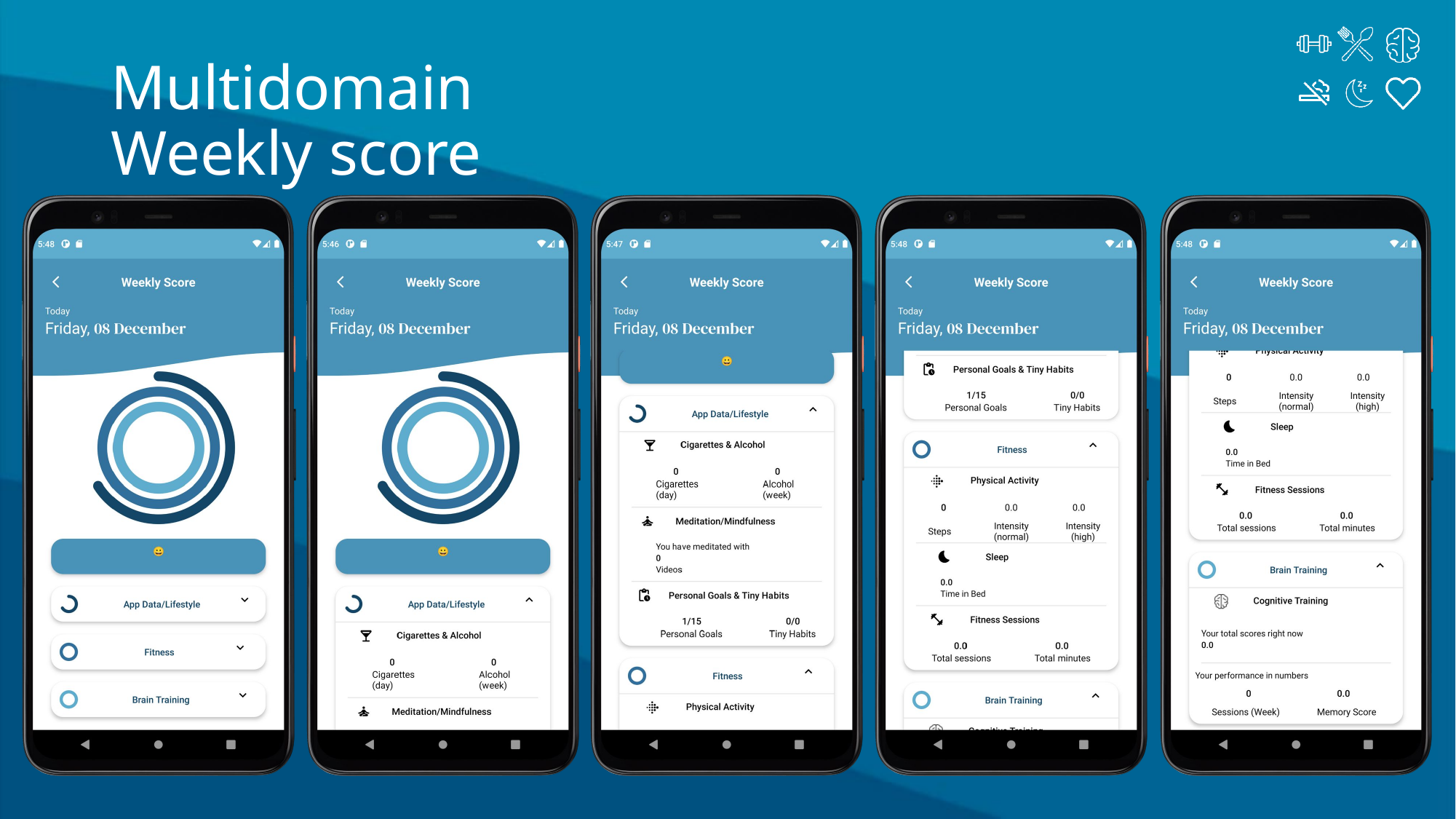

# Multidomain Weekly score
