# Supplementary material for: Design of a Mobile App and a Clinical Trial Management System for Cognitive Health and Dementia Risk Reduction: User-Centered Design Approach
Source: JMIR Aging. 2025 Jul 2;8:e66660. doi: 10.2196/66660 (PMC12268216; doi:10.2196/66660)
Supplement: Multimedia Appendix 6 [file aging_v8i1e66660_app6.pptx]

## Slide 1
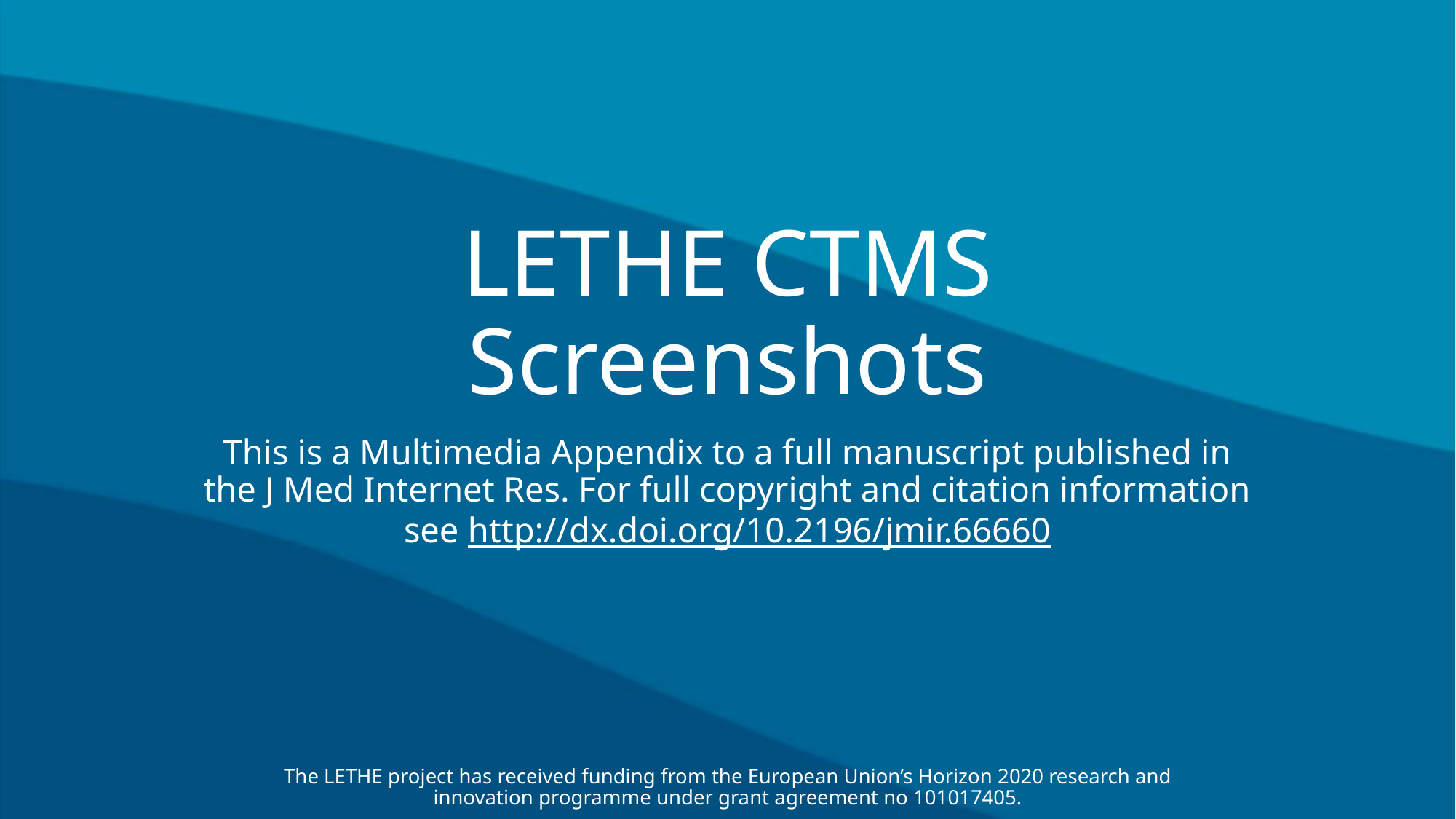

# LETHE CTMS Screenshots
This is a Multimedia Appendix to a full manuscript published in the J Med Internet Res. For full copyright and citation information see http://dx.doi.org/10.2196/jmir.66660
The LETHE project has received funding from the European Union’s Horizon 2020 research and innovation programme under grant agreement no 101017405.

## Slide 2
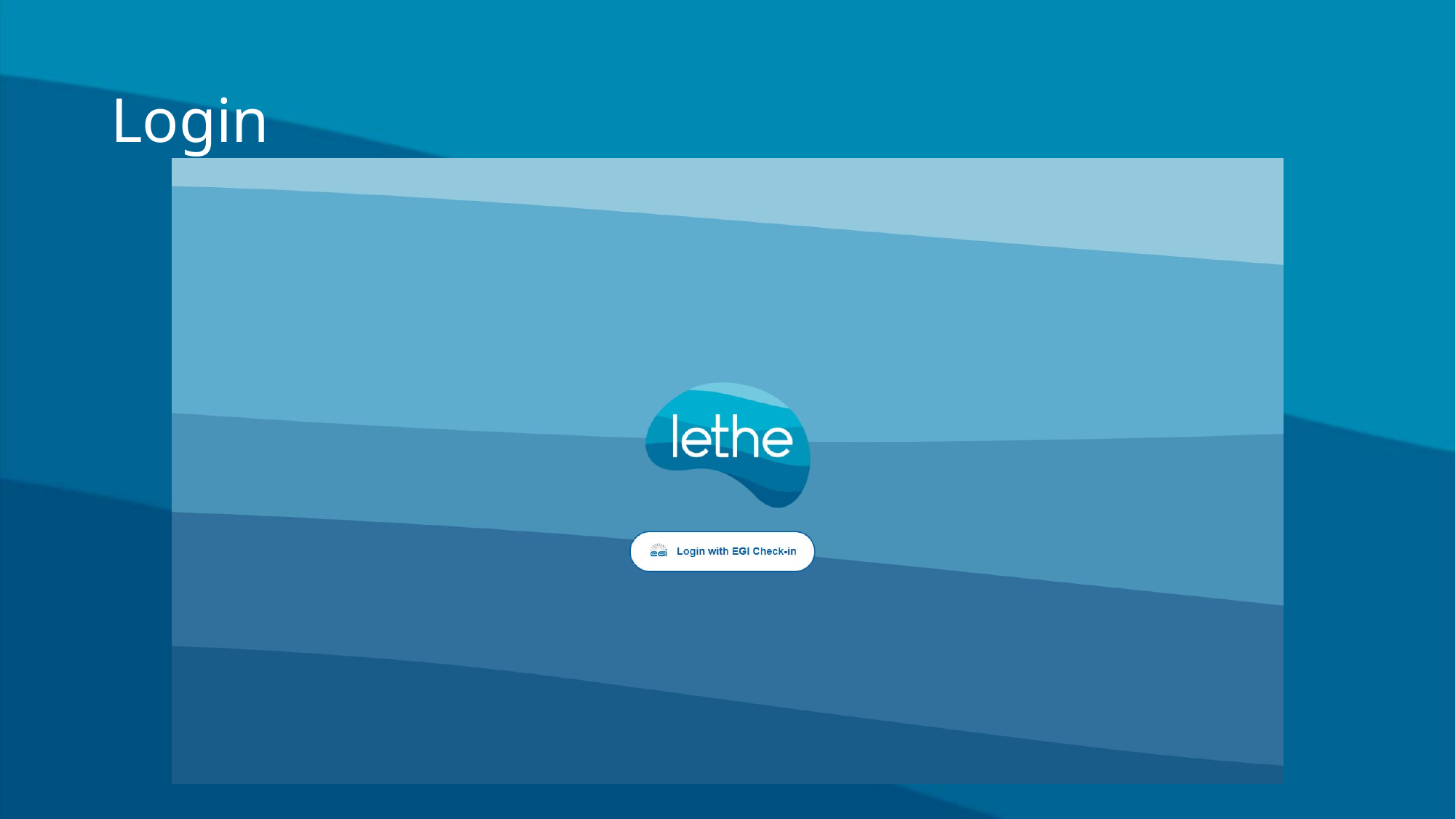

# Login

## Slide 3
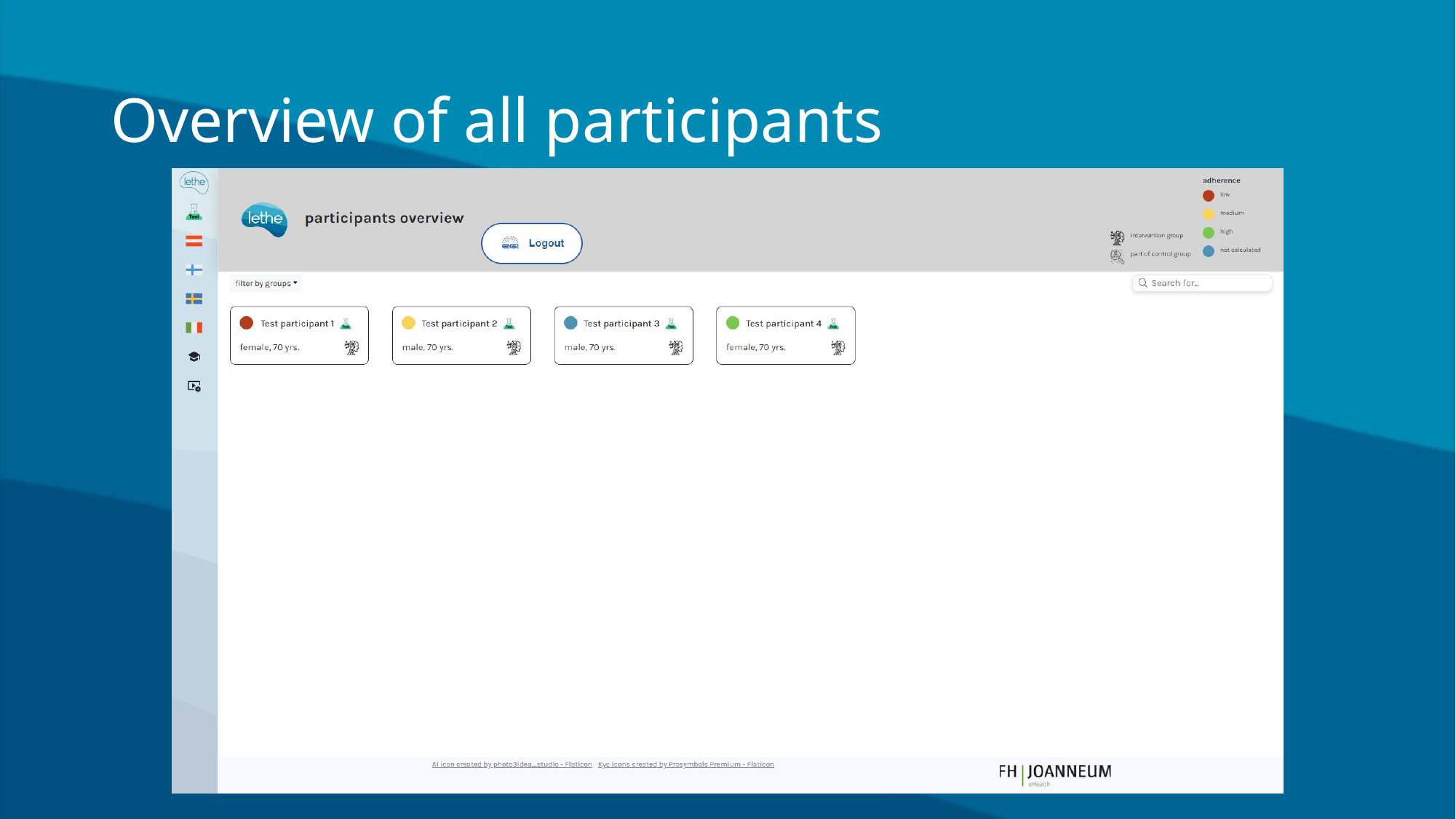

# Overview of all participants

## Slide 4
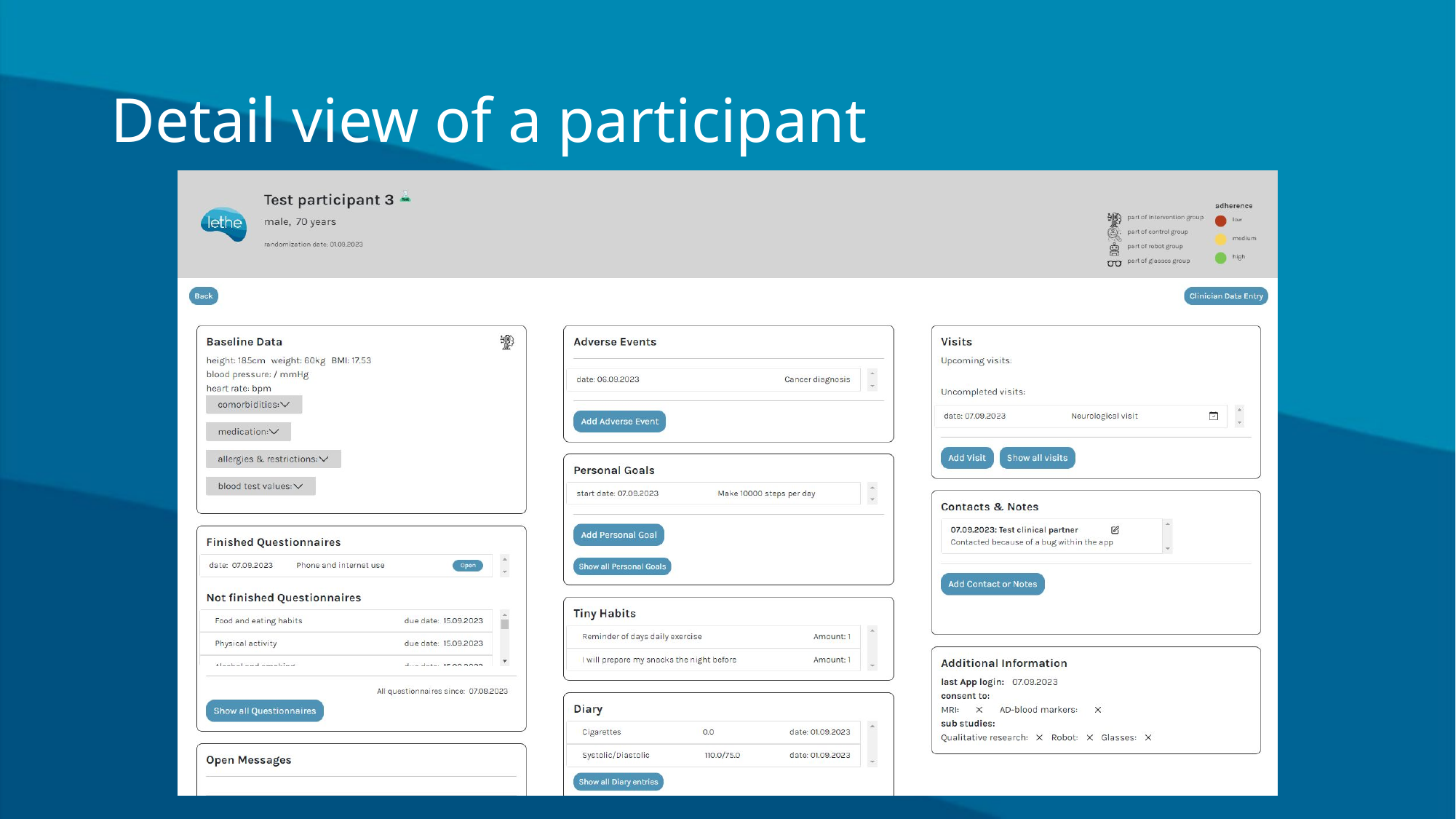

# Detail view of a participant

## Slide 5
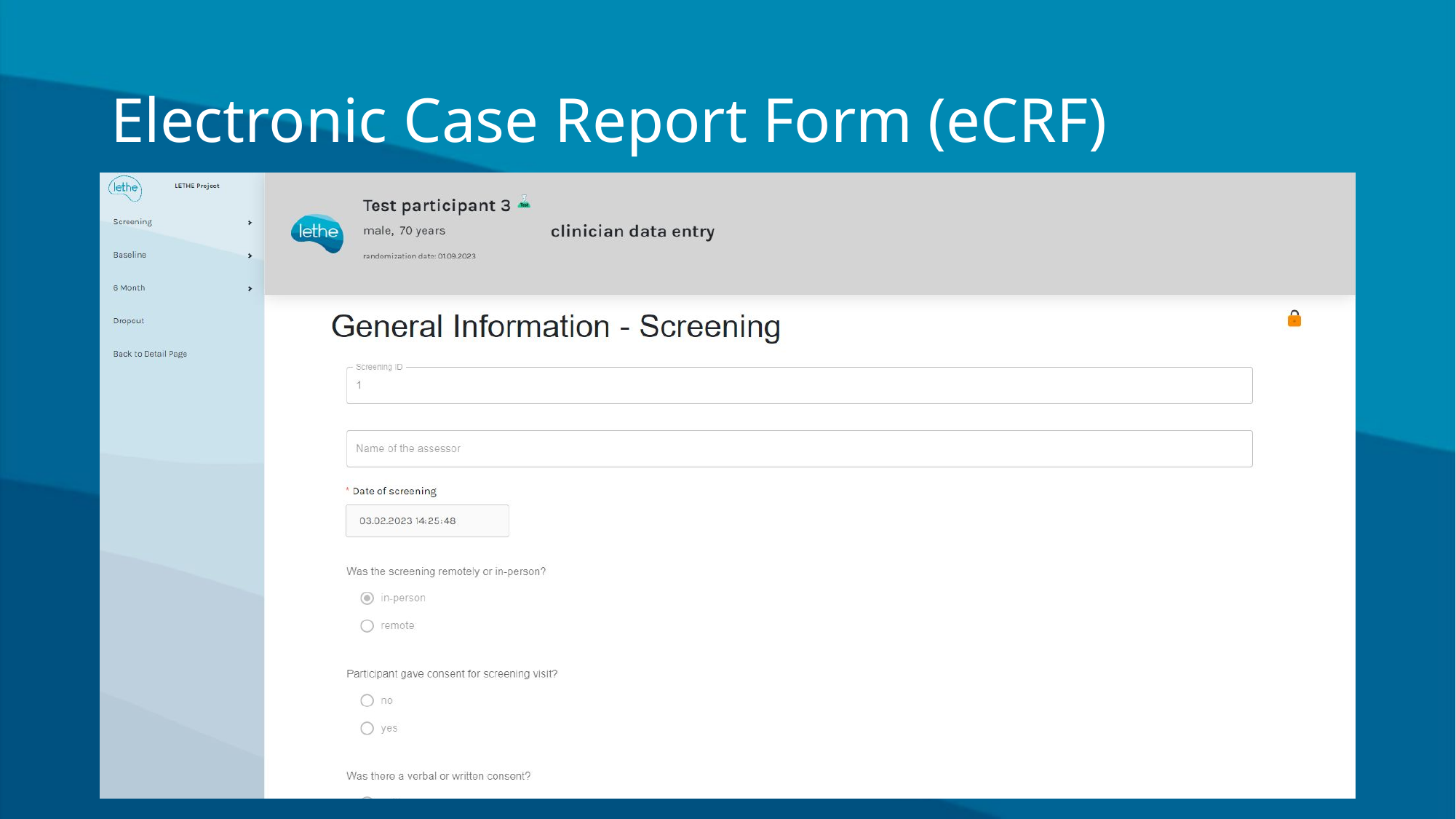

# Electronic Case Report Form (eCRF)
